# Mutations in the promoter, intron and CDS of two *FAD2* generate multiple alleles modulating linoleic acid level in yellow mustard

**DOI:** 10.1038/s41598-017-08317-y

**Published:** 2017-08-15

**Authors:** Fangqin Zeng, Vicky Roslinsky, Bifang Cheng

**Affiliations:** Agriculture and Agri-Food Canada, Saskatoon Research and Development Centre, 107 Science Place, Saskatoon, SK S7N 0X2 Canada

## Abstract

Linoleic acid (C18:2) is an important polyunsaturated fatty acid in the seed oil of many crops. Here, we report that mutations in the promoter, intron and CDS of the *FAD2* genes *SalFAD2*.*LIA*1 and *SalFAD*2.*LIA*2 generate three alleles *LIA* ^1*a*^, *LIA* ^*1b*^ and *lia* ^*1*^ and two alleles *LIA* ^2^ and *lia* ^2^, respectively, controlling the C18:2 variation (4.4–32.7%) in yellow mustard. The allelic effect on increasing C18:2 content is *LIA* ^*1a*^ > *LIA* ^*1b*^ > *lia* ^*1*^, *LIA* ^2^ > *lia* ^2^, and *LIA* ^*1a*^ > *LIA* ^2^. The five *FAD* *2* alleles each contain two exons, one intron and a promoter adjacent to exon 1. *LIA* ^1*a*^ has a 1152 bp CDS, a 1221 bp intron with promoter function and a 607 bp promoter. Compared with *LIA* ^*1a*^, the intron of *LIA* ^*1b*^ has reduced promoter activity and that of *LIA* ^2^ and *lia* ^2^ has no promoter function due to extensive SNP and indel mutations. *lia* ^1^ differed from *LIA* ^1*b*^ by having an insertion of 1223 bp retrotransposon in its intron. *lia* ^*2*^ with mutations in the promoter has reduced promoter activity compared with *LIA* ^*2*^. This study revealed that complex quantitative variation of trait phenotype in plants could be modulated by multiple alleles of oligogenic loci resulting from mutations in the regulatory region and CDS.

## Introduction

Polyunsaturated fatty acids including linoleic (C18:2) and linolenic (C18:3) acids are the major components of seed storage lipids in higher plants and important factors in determining the qualities of edible oil such as oxidative stability^1^ and nutritional value^2^. Biosynthesis of linoleic acid is catalyzed by the microsomal delta-12 fatty acid desaturase (FAD2) localized in the endoplasmic reticulum^3^. The *FAD2* gene in *Arabidopsis thaliana* was isolated from mutants created by T-DNA insertions^4^. Subsequent investigations have identified one or more *FAD2* copies in various crops such as cabbage (*Brassica oleracea*) and turnip (*B*. *rapa*)^5^, rapeseed (*B*. *napus*)^6^, cotton (*Gossypium hirsutum*)^7^, sesame (*Sesamum indicum*)^8^, olive (*Olea europaea*)^9^ and grape (*Vitis labrusca*)^10^. The *FAD2* gene contains two exons, one intron embedded within the 5′ UTR and a promoter^5–7, 10, 11^. The intron of *FAD2* has promoter function and can regulate the expression level of *FAD*2^6, 11^. The protein encoded by *FAD2* contains six transmembrane domains and three histidine boxes (H box) harboring eight iron-binding histidines in *A*. *thaliana* ^4^. The histidines appear to be crucial for proper enzymatic function, since substitution of a histidine with a different amino acid disrupts desaturase function^12, 13^.

It has been reported that mutations in the coding DNA sequence (CDS) of *FAD2* are correlated with the increase of oleic acid (C18:1) content in *B*. *napus* ^14–16^ and *B*. *rapa* ^17^. The high oleic acid (~77%) variant DMS100, developed through ethyl methane sulfonate (EMS) mutagenesis, was due to a single nucleotide mutation that resulted in the occurrence of a stop codon (TAG) leading to premature termination of the open reading frame of *FAD2* in *B*. *napus* ^14^. However, only partial coding DNA sequences (nucleotide 148–1128) of the *FAD2* were cloned from DMS 100 and the wild-type line Quantum in this study. Yang *et al*. (2012) successfully cloned four copies of *FAD*2 genes from the high C18:1 (~78%) variant SW Hiskory and the wild-type line JA177 in *B*. *napus*. Sequence alignment indicated that a 4 bp insertion at the position 567–568th base pair of the *FAD2* gene *BnaA*.*FAD2*.*a* was responsible for the high C18:1content in SW Hiskory^15^. Interestingly, *BnaA*.*FAD2*.*a* of the high C18:1 (~75%) variant Cabriolet was also non-functional, but resulted from a 1 bp deletion, leading to a frame shift and a truncated protein in *B*. *napus* ^16^. The high C18:1 content of line Jo4072 could be resulted from the transition at nucleotide 484 in the CDS of *FAD2* in *B*. *rapa* ^17^. It is worth-noting that the function of the encoded enzyme, the transcriptional level and the promoter activity of different *FAD2* alleles were not characterized and the introns of *FAD2* were not found in these studies.

Condiment yellow mustard (*Sinapis alba* L.) is phylogenetically related to *Brassica* species^18^ and is an obligate outcrossing crop. Open-pollinated (OP) population varieties of this crop comprise great genetic variation. Various plant morphological types, fatty acid and glucosinolate variants have been isolated via inbreeding of the OP varieties in yellow mustard^19–21^. The molecular mechanism underlying the natural occurring erucic variants in yellow mustard is revealed to be different from that reported in *B*. *napus*. The erucic acid variants have resulted from SNP/indel mutations in the CDS of fatty acid elongation 1 (*FAE*1) in *B*. *napus* ^22–26^. However, transposable element insertions and epigenetic modification in the *FAE1* had led to the occurrence of multi-alleles in yellow mustard^27^. Linoleic acid (C18:2) content ranged from 6.2% to 14.2% in yellow mustard germplasm^28^. Recently, three lines Y1798, Y514 and Y1801 with low (average: 4.2%, range: 3.5–5.3%), medium (average: 12.5%, range: 12.0–13.0%) and high (average: 31.5%, range: 28.7–37.7%) C18:2 contents, respectively, have been successfully developed in this species^29, 30^. In the present study, the CDS, promoter and intron of *FAD*2 from each of the three lines Y1801, Y514 and Y1798 have been molecularly and functionally characterized. Here, we report that mutations in the promoter, intron and CDS of two *FAD*2 generate multiple alleles modulating the quantitative variation of C18:2 content in yellow mustard.

## Results

### Mapping QTLs for C18:2 content

The average C18:2 contents of the F_1_ seeds derived from the crosses of Y1798 (low) × Y1801 (high) and Y1798 (low) × Y514 (medium) were 10.2% and 6.9%, which were significantly lower than the mid-parent values of 18.6% (t = 9.80, *p* < 0.01) and 8.8% (t = 2.60, *p* < 0.01), respectively (Table 1; Supplementary Fig. S1), suggesting a partial dominance of the low over both high and medium C18:2 contents. The F_1_ seeds of Y1801 (high) × Y514 (medium) had an average C18:2 content of 21.4% which was similar with the mid-parent value of 23.0% (t = 0.93, *p* = 0.37) (Table 1; Supplementary Fig. S1). The F_2_ seeds of each of the three crosses showed a continuous frequency distribution in the C18:2 content (Supplementary Fig. S2) and were not possible to be classified into discrete groups. Therefore, QTL mapping was used to identify the QTLs controlling C18:2 content.

**Table 1:** Fatty acid profile of the parental lines Y1798, Y514 and Y1801, F_1_ seeds, and mid-parent value in yellow mustard.

Parental lines	Generation	C18:1 (% of total fatty acids)	C18:2 (% of total fatty acids)	C18:3 (% of total fatty acids)	C22:1 (% of total fatty acids)
Y1798	S_4_	27.3 ± 2.3	4.2 ± 0.1	13.0 ± 0.4	36.3 ± 2.4
Y514	DH	67.6 ± 0.5	12.5 ± 0.2	10.8 ± 0.5	0.2 ± 0.1
Y1801	S_5_	21.7 ± 0.7	31.5 ± 0.9	12.5 ± 0.5	16.1 ± 0.3
Y1798 (low) × Y1801 (high)	F_1_	29.3 ± 0.6	10.2 ± 0.2**^1^	14.7 ± 0.4	26.2 ± 0.3
Mid-parent value of Y1798 and Y801		24.5	18.6	12.8	26.2
Y1798 (low) × Y514 (medium)	F_1_	32.0 ± 0.4	6.9 ± 0.2**^2^	13.3 ± 0.2	26.6 ± 0.3
Mid-parent value of Y1798 and Y514		47.8	8.8	11.9	18.3
Y1801 (high) × Y514 (medium)	F_1_	30.2 ± 0.7	21.4 ± 0.4	17.8 ± 0.3	11.1 ± 0.2
Mid-parent value of Y1801 and Y514		44.7	23.0	11.7	8.2

Fatty acid content is expressed as mean value ± standard deviation.

**Statistical significant difference at *p* = 0.01 level. ^1^Comparison between F_1_ and mid-parent value of Y1798 and Y1801; ^2^Comparison of F_1_ and mid-parent value of Y1798 and Y514.

One hundred thirty, one hundred ten and one hundred fourteen polymorphic ILP markers and the allele-specific markers for the *FAE1* gene and the *FAD3* genes *SalFAD3*.*LA1* and *SalFAD3*.*LA*2 were used to genotype individual plants of the F_2_ populations of Y1798 (low) × Y1801 (high), Y1798 (low) × Y514 (medium) and Y1801 (high) × Y514 (medium). Twelve linkage groups were constructed using the polymorphic markers genotyped in each of the three crosses. Based on the common ILP markers, the 12 linkage groups corresponded to Sal01 to Sal12 of the constructed *S*. *alba* map by Javidfar and Cheng (2013)^31^. The *FAE1* gene was mapped to Sal03 and the *FAD3* genes *SalFAD3*.*LA1* to Sal02 and *SalFAD3*.*LA*2 to Sal10 (Supplementary Fig. S3A–C).

**Figure 1:**
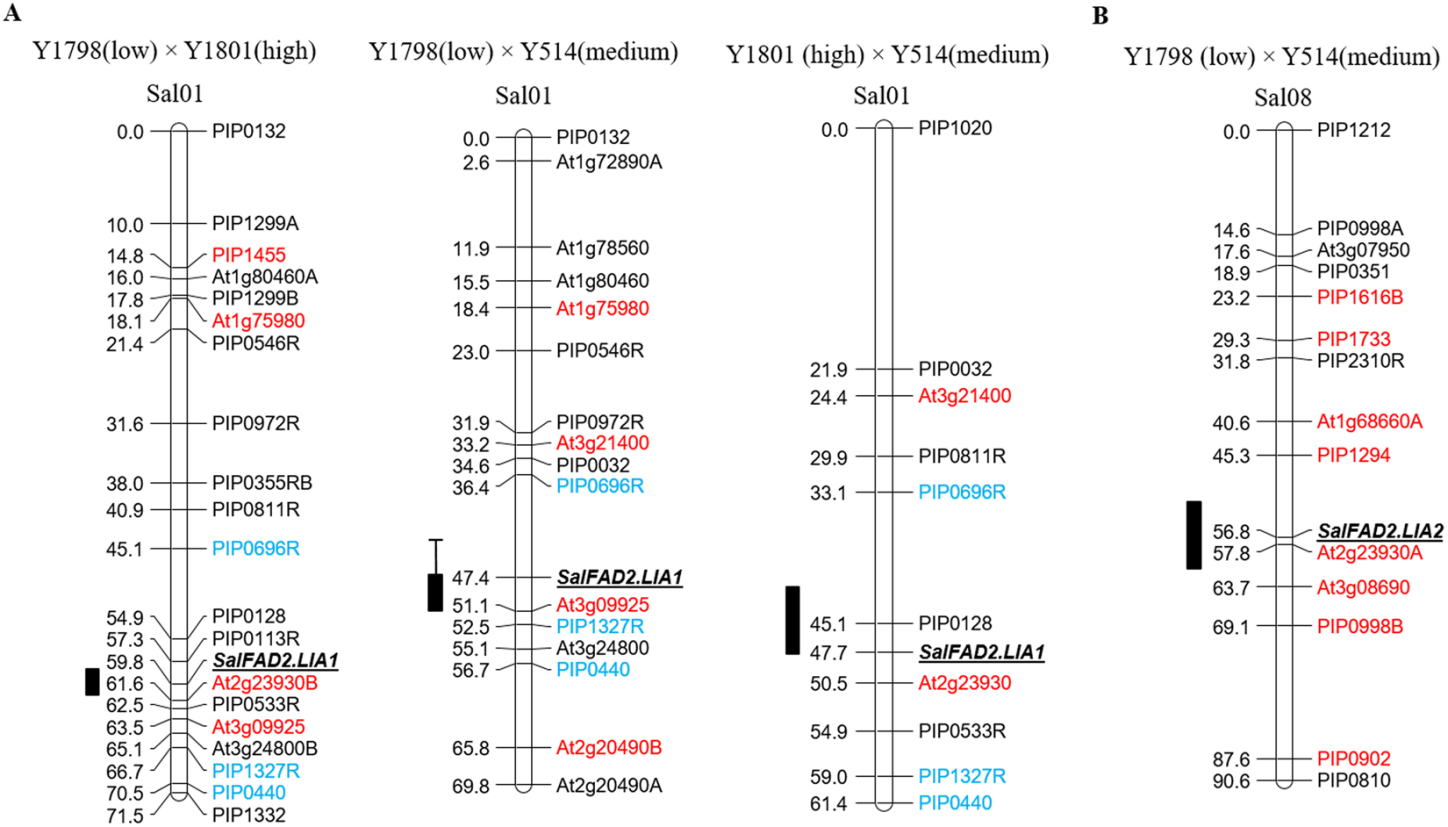
Mapping QTLs controlling C18:2 content in yellow mustard. (**A**) The C18:2 QTL in Sal01 is located between PIP0113R and At2g23930B in Y1798 (low) × Y1801 (high), between PIP0696R and At3g09925 in Y1798 (low) × Y514 (medium), and between PIP0696R and At2g23930 in Y1801 (high) × Y514 (medium). (**B**) The C18:2 QTL in Sal08 is located between PIP1294 and At3g08690 in Y1798 (low) × Y514 (medium). 1-LOD and 2-LOD supporting intervals of each C18:2 QTL were marked by thick and thin bars, respectively. Markers in blue font were detected in all three crosses. Markers in red font were mapped to the same linkage groups as reported by Javidfar and Cheng (2013)^31^. The *SalFAD2*.*LIA1* and *SalFAD2*.*LIA2* genes co-localized with their C18:2 QTL peaks in the linkage groups Sal01 and Sal08, respectively.

Three QTLs for C18:2 content, two QTLs for C18:3 content and one QTL for C22:1 content were identified in the F_2_ population of Y1798 (low) × Y1801 (high) (Fig. 1, Supplementary Table 2 and Supplementary Fig. S3A). The three C18:2 QTLs explained 43.2%, 42.8% and 5.6% of the phenotypic variation and mapped to the linkage groups Sal01, Sal02 and Sal03, respectively. The C18:2 QTL (LOD = 40.63) on Sal01 was located between the markers PIP0113R and At2g23930B (Fig. 1A). The C18:2 QTL (LOD = 40.44) on Sal02 co-localized with the QTL for C18:3 content and the *FAD3* gene *SalFAD3*.*LA1* (Supplementary Fig. S3A). The C18:2 QTL (LOD = 9.99) on Sal03 shared the same region with the C22:1 QTL and the *FAE1* gene (Supplementary Fig. S3A). In the F_2_ population of Y1798 (low) × Y514 (medium), three C18:2 QTLs, three C18:3 QTLs and one C22:1 QTL were detected. The three C18:2 QTLs, responsible for 47.3%, 24.4% and 9.5% of the phenotypic variation, were assigned to Sal01, Sal03 and Sal08, respectively (Fig. 1, Supplementary Table 2 and Supplementary Fig. S3B). The C18:2 QTL (LOD = 25.44) on Sal01 was located between the markers PIP0696R and At3g09925 (Fig. 1A). The C18:2 QTL on Sal03 co-localized with the C22:1 QTL and the *FAE1* gene (Supplementary Fig. S3B). The C18:2 QTL (LOD = 3.47) on Sal08 was located between the markers PIP1294 and At3g08690 (Fig. 1B). Two C18:2 QTLs, two C18:3 QTLs and one C22:1 QTL were revealed in the F_2_ population of Y1801 (high) × Y514 (medium) (Fig. 1, Supplementary Table 2 and Supplementary Fig. S3C). The C18:2 QTL (LOD = 14.40), accounting for 42.6% of the C18:2 content variation, was mapped to the same region as the C18:3 QTL and *FAD3* gene *SalFAD3*.*LA1* on Sal02 (Supplementary Fig. S3C). To eliminate the confounding effect of C18:3 variation, 22 F_2_ plants carrying the dominant homozygous *SalFAD3*.*LA1* alleles *LA* ^*1*^ *LA* ^*1*^ were removed from the F_2_ population. QTL analysis using the remaining 102 F_2_ plants identified one C18:2 QTL (LOD = 3.54) on Sal01, responsible for 14.9% of the C18:2 content variation (Fig. 1A) in addition to the C18:3 QTL on Sal02. This indicated that the effect of the C18:3 QTL on C18:2 content is larger than the C18:2 QTL.

The C18:2 QTL on Sal02 co-localized with the *FAD3* gene *SalFAD3*.*LA1* and that on Sal03 with the *FAE1* gene. Therefore, only the C18:2 QTLs on Sal01 and Sal08 implied the presence of *FAD*2 genes. The *FAD*2 gene on Sal01 was referred to as *SalFAD2*.*LIA*1, while that on Sal08 as *SalFAD2*.*LIA2*. The C18:2 QTL on Sal01 was detected in the three crosses Y1798 (low) × Y1801 (high), Y1798 (low) × Y514 (medium) and Y1801 (high) × Y514 (medium), indicating that Y1801, Y514 and Y1798 carried different alleles, designated as *LIA* ^1*a*^, *LIA* ^1*b*^ and *lia* ^*1*^, respectively, at the *SalFAD*2.*LIA1* locus. The C18:2 QTL on Sal08 was identified in the cross of Y1798 (low) × Y514 (medium), but not in Y1801 (high) × Y514 (medium), suggesting that Y1801 and Y514 harboured the same allele, designated as *LIA* ^*2*^, while Y1798 had the recessive allele *lia* ^*2*^ at the *SalFAD*2.*LIA*2 locus. Thus, the C18:2 genotypes of Y1801, Y514 and Y1798 were *LIA* ^*1a*^ *LIA* ^*1a*^ *LIA* ^2^ *LIA* ^*2*^, *LIA* ^*1b*^ *LIA* ^*1b*^ *LIA* ^2^ *LIA* ^*2*^ and *lia* ^1^ *lia* ^*1*^ *lia* ^2^ *lia* ^*2*^, respectively.

### Cloning of the *SalFAD2.LIA1* alleles *LIA*^1*a*^, *LIA*^1*b*^ and *lia*^*1*^ and the *SalFAD2.LIA2* alleles *LIA*^*2*^ and *lia*^*2*^

**Figure 2:**
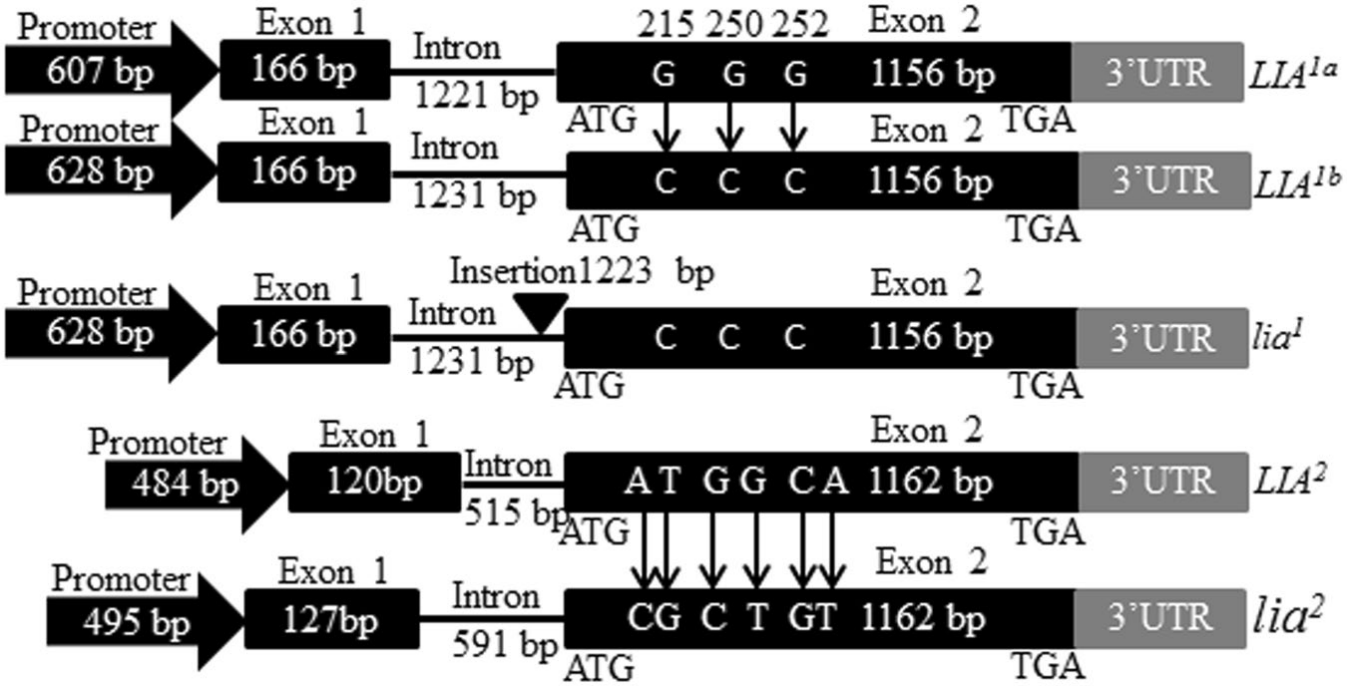
Schematic representation of the structure of the *SalFAD2*.*LIA1* alleles *LIA* ^*1a*^, *LIA* ^*1b*^, *lia* ^*1*^ and the *SalFAD2*.*LIA2* alleles *LIA* ^2^ and *lia* ^2^. Black rectangle: Exon; Straight line: Intron; Solid black arrow: Promoter; Grey rectangle: 768 bp 3′ UTR; Vertical long arrow: Point mutation; Inverted triangle: Insertion.

The coding DNA sequnce (CDS) of *LIA* ^1*a*^ and *LIA* ^2^ were sucessfully cloned from Y1801 (*LIA* ^1*a*^ *LIA* ^*1a*^ *LIA* ^2^ *LIA* ^2^), while the CDS of *LIA* ^1*b*^ and *LIA* ^2^ from Y514 (*LIA* ^1*b*^ *LIA* ^1*b*^ *LIA* ^*2*^ *LIA* ^2^) and the CDS of *lia* ^*1*^ and *lia* ^2^ from Y1798 (*lia* ^1^ *lia* ^*1*^ *lia* ^2^ *lia* ^2^) using primer pair No 1 (Supplementary Table S1; Supplementary Fig. S4). *LIA* ^1*a*^, *LIA* ^1*b*^ and *lia* ^*1*^ had a CDS of 1152 bp encoding a polypeptide of 383 amino acids. The CDS of *LIA* ^1*b*^ and *lia* ^*1*^ shared the same nucleotide sequence. Sequence alignment with *LIA* ^1*a*^ allowed the identification of fifteen point mutations at positions 99, 156, 171, 215, 250, 252, 396, 534, 573, 615, 681, 684, 735, 957 and 1032 in the CDS of *LIA* ^1*b*^ and *lia* ^*1*^. The mutations at positions 215, 250 and 252 (G of *LIA* ^1*a*^ to C of *LIA* ^1*b*^ and *lia* ^*1*^) led to the two amino acid changes: the threonine and phenylalanine residues at position 72 and 84 in the protein encoded by *LIA* ^1*a*^ were substituted by the serine and valine residues in the protein encoded by *LIA* ^1*b*^ and *lia* ^*1*^ (Fig. 2; Supplementary Fig. S5). The cloned CDS of *LIA* ^2^ from Y1801 and Y514 were identical in size and nucleotide sequence. *LIA* ^2^ and *lia* ^2^ had a CDS of 1158 bp encoding a polypeptide of 385 amino acids. Twelve point mutations at positions 34, 78, 125, 174, 187, 363, 447, 450, 506, 597, 616 and 642 were observed in the CDS of *lia* ^2^ when compared with that of *LIA* ^2^. The mutations at positions 34, 125, 174, 187, 506, and 642 caused amino acids changes; the threonine, proline, cysteine, serine, glycine and asparagine residues at positions 12, 42, 58, 63, 169 and 206 in the protein sequence encoded by *LIA* ^2^ were substituted by serine, histidine, tryptophan, alanine, alanine and aspartic acid residues in the protein encoded by *lia* ^2^ (Supplementary Fig. S5).

Sequence alignment of the CDS of *LIA* ^1*a*^ with that of *LIA* ^2^ indicated that *LIA* ^1*a*^ harboured a 3 bp deletion at position 516 and a 3 bp deletion at position 613, which resulted in the loss of the asparagine residue at position 175 and the tyrosine residue at position 204 in the polypeptide (Supplementary Fig. S5). In addition, 104 SNPs were observed in the CDS of *LIA* ^1*a*^ and *LIA* ^2^, of which 27 SNPs led to amino acid changes in the first, second, third, fifth and sixth transmembrane domains (Supplementary Fig. S5).

DNA fragments comprising the 736 bp downstream sequences from the translation stop codon were cloned for *LIA* ^1*a*^, *LIA* ^1*b*^, *lia* ^*1*^, *LIA* ^2^ and *lia* ^2^, respectively, using primer pair No 2 (Supplementary Table S1; Supplementary Fig. S4). These DNA fragments had the same nucleotide sequence.

The 5′ flanking sequences of *LIA* ^1*a*^, *LIA* ^1b^ and *lia* ^*1*^ were cloned by two rounds of PCR walking using primer pairs No 3 and No 4 (Supplementary Table S1; Supplementary Fig. S4). The cloned 5′ flanking fragments of *LIA* ^1*a*^, *LIA* ^1*b*^ and *lia* ^*1*^ were 671 bp, 681 bp and 1909 bp, respectively. BLAST analysis of the cloned upstream sequences of *LIA* ^1*a*^, *LIA* ^1*b*^ and *lia* ^*1*^ against the NCBI nucleotide sequence database revealed that they had high similarity to the intron of *BnFAD*2 (KF038144) in *B*. *napus* with the E value being 7e^−105^, 3e^−77^ and 3e^−81^, respectively. Therefore, the promoter sequence of *BnFAD*2 (KF038144) was used to design primer pair No 5 (Supplementary Table S1; Supplementary Fig. S4) to clone the promoters for *LIA* ^1*a*^, *LIA* ^1*b*^ and *lia* ^*1*^. The cloned promoter of *LIA* ^1*a*^ was 607 bp, while those of *LIA* ^1*b*^ and *lia* ^*1*^ were 628 bp. The gap between the promoter region and the 5′ flanking fragment was filled with a 720 bp fragment for *LIA* ^1*a*^, a 724 bp fragment for *LIA* ^1*b*^ and a 719 bp fragment for *lia* ^*1*^, obtained using primer pair No 6 (Supplementary Table S1; Supplementary Fig. S4). Sequence alignments of the putative promoter and the 5′ flanking fragments obtained using primer pairs No 3, 4, 5 and 6 revealed that the cloned 5′ upstream sequences of *LIA* ^1*a*^, *LIA* ^1*b*^ and *lia* ^*1*^ are 1998 bp, 2019 bp and 3252 bp, respectively.

The 5′ flanking sequences of *LIA* ^2^ and *lia* ^2^ were cloned by two rounds of PCR walking using primer pairs No 7 and No 8 (Supplementary Table S1; Supplementary Fig. S4). The cloned 5′ flanking fragments of *LIA* ^2^ from Y1801 and Y514 were 1123 bp and identical in sequence, while that of *lia* ^2^ from Y1798 was 1585 bp. BLAST analysis of the 1123 bp upstream sequence of *LIA* ^2^ and the 1585 bp upstream sequence of *lia* ^2^ against the NCBI nucleotide sequence database revealed that they had high similarity with the promoter, first exon and intron sequences of *BjFAD2-*1 (HM147243) in *B*.* juncea* with the E value of 3e^−15^ and 9e^−11^, respectively.

Thus, the entire nucleotide sequence (5′ upstream and coding regions) for each of the *FAD*2 alleles *LIA* ^1a^, *LIA* ^*1b*^, *lia* ^*1*^, *LIA* ^2^ and *lia* ^*2*^ with sizes of 3150 bp, 3181 bp, 4404 bp, 2281 bp and 2744 bp, respectively, was obtained by PCR walking. To validate these results, *LIA* ^1*a*^, *LIA* ^*1b*^ and *lia* ^*1*^ were each cloned in its entirety using primer pair No 9, while *LIA* ^2^ and *lia* ^2^ using primer pair No 10 (Supplementary Table S1). As expected, the resulting DNA fragments of the *SalFAD2*.*LIA*1 and *SalFAD2*.*LIA2* alleles had the same sizes (Supplementary Fig. S6) and nucleotide sequences as those obtained from PCR walking.

### The *SalFAD2*.*LIA1* and *SalFAD2*.*LIA2* alleles differ in their promoter, exons 1 and 2, and intron

Sequence alignment with *BnFAD2* (KF038144) from *B*. *napus* indicated that the cloned 5′ upstream sequences of *LIA* ^*1a*^, *LIA* ^*1b*^ and *lia* ^*1*^ comprised the first exon. A 1322 bp cDNA fragment was thus sucessfully cloned for *LIA* ^*1a*^, *LIA* ^*1b*^ and *lia* ^1^ using primer pair No 11 (Supplementary Table S1) designed based on the sequences of their deduced first exon and cloned CDS. Sequence alignment of the cDNA and genomic DNA sequences indicated that *LIA* ^1*a*^, *LIA* ^1*b*^ and *lia* ^1^ each comprised a promoter, exon 1, an intron and exon 2, but differed in size and nucleotide sequence (Fig. 2). The promoter, exon 1, intron and exon 2 of *LIA* ^1*a*^ were 607 bp, 166 bp, 1221 bp and 1156 bp, respectively. *LIA* ^1*b*^ and *lia* ^1^ shared the same promoter with 628 bp and the first exon of 166 bp and the second exon of 1156 bp. The intron of *lia* ^1^ was different from that (1231 bp) of *LIA* ^1*b*^ by having a 1223 bp insertion at position −26 bp in the 5′ upstream region from the translation start codon (Fig. 2; Supplementary Fig. S7). Sequence alignment with the promoter of *LIA* ^*1a*^ revealed that the promoter sequences of *LIA* ^*1b*^ and *lia* ^*1*^ harboured insertions of 12 bp, 4 bp, 4 bp, 1 bp, 1 bp and 3 bp at position 25, 171, 392, 494, 522 and 548, and deletions of 3 bp and 1 bp at positions 66 and 369 as well as 9 point mutations at positions 57, 134, 135, 337, 353, 404, 491, 534 and 546 (Supplementary Fig. S8A). Exon 1 of *LIA* ^*1b*^ and *lia* ^*1*^ harboured a 2 bp deletion at position 34 and a 2 bp insertion at position 153 as well as three point mutations at positions 25, 82 and 106 in comparison with that of *LIA* ^*1a*^ (Supplementary Fig. S8B). The exon 2 of *LIA* ^*1a*^, *LIA* ^*1b*^ and *lia* ^*1*^ contained the CDS of 1152 bp. Sequence alignment with the intron of *LIA* ^*1a*^ revealed that the intron of *LIA* ^*1b*^ harboured insertions of 5 bp, 1 bp, 1 bp, 1 bp, 5 bp, 5 bp and 3 bp at positions 50, 71, 771, 830, 877, 1142 and 1192, and deletions of 1 bp at positions 135, 200, 271, 373, 421, 956, 1046 and 1087, a 3 bp deletion at position 445 as well as 49 point mutations (Supplementary Fig. S9). The 1223 bp insertion in the intron of *lia* ^*1*^ contained a coding region of 1044 bp that has a primer binding site (PBS) (Supplementary Fig. S10A) and encode a protein with 348 amino acids (Supplementary Fig. S10B). The protein contained the conserved domain of gag-polypeptide of LTR copia-type retrotransposon and exhibited 65% identity with putative reverse transcriptase (AAD11595) in *Arabidopsis* (E = 4e^−103^). Therefore, the 1223 bp insertion in the intron of *lia* ^*1*^ appears to be a retrotransposon.

Sequences alignment with *BjFAD*2*-*1 (HM147243) from *B*. *juncea* indicated that the cloned 5′ upsteam sequences of the *SalFAD*2.*LIA*2 alleles *LIA* ^2^ and *lia* ^2^ contained the first exon. The cDNA sequences of 1282 bp and 1289 bp for *LIA* ^2^ and *lia* ^2^, respectively, were cloned using primer pair No 12 (Supplementary Table S1) designed based on the deduced first exon and the cloned CDSs. Alignment of the cDNA and genomic DNA sequences indicated that *LIA* ^2^ contained a promoter of 484 bp, the first exon of 120 bp, the second exon of 1162 bp and an intron of 515 bp, while *lia* ^2^ had a promoter of 495 bp, the first exon of 127 bp, the second exon of 1162 bp and an intron of 591 bp (Fig. 2). Sequences alignment with *LIA* ^2^ revealed that *lia* ^2^ harboured deletions of a 2 bp, 4 bp, 1 bp, 2 bp and 1 bp at positions 2, 184, 222, 274 and 420, and insertions of 1 bp, 1 bp, 7 bp, 10 bp and 1 bp at positions 312, 339, 353, 368 and 434 in the promoter (Supplementary Fig. S11A), and a 5 bp and a 2 bp insertion at positions 32 and 47 in the first exon (Supplementary Fig. S11B). The exon 2 of *LIA* ^2^ and *lia* ^2^ comprised the CDS of 1158 bp that differed in sequence (Fig. 2). The intron of *lia* ^*2*^ harboured 9 bp, 2 bp, 1 bp, 2 bp, 1 bp and 14 bp deletions at positions 76, 141, 154, 203, 532 and 546, and 17 bp, 9 bp, 17 bp, 15 bp, 9 bp, 29 bp, 8 bp and 1 bp insertions at positions 247, 276, 297, 381, 399, 413, 474 and 594, respectively, compared with the intron of *LIA* ^*2*^ (Supplementary Fig. S11C). Compared with *LIA* ^1*a*^, *LIA* ^*2*^ harboured 103 SNPs, 5 insertions and 24 deletions in the promoter and 106 SNPs, one 5 bp insertion and 43 deletions in the intron (Supplementary Fig. S12).

### Heterologous expression of the *SalFAD2*.*LIA*1 and *SalFAD2*.*LIA2* alleles in yeast

**Figure 3:**
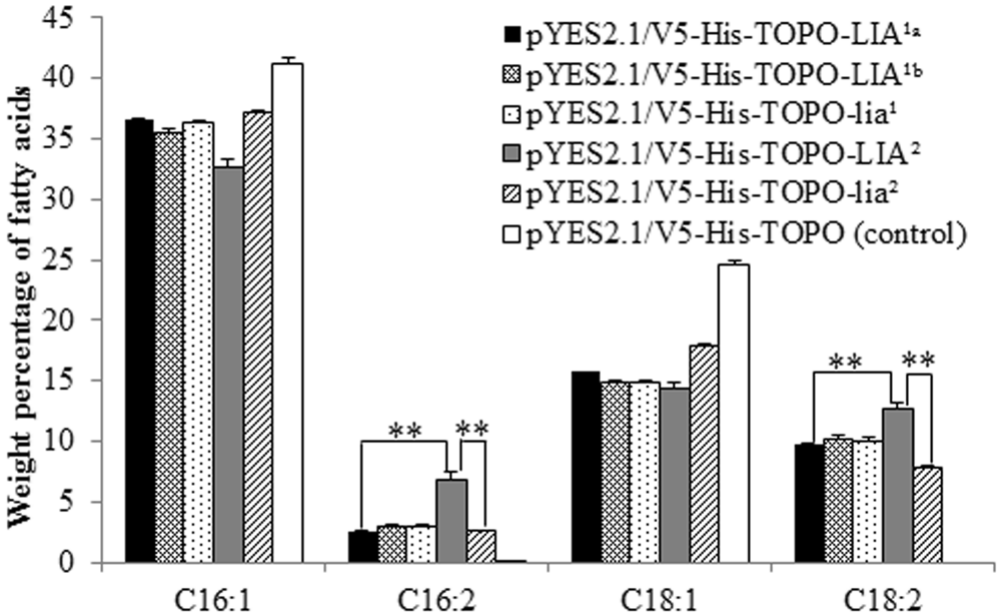
Heterologous expression of the *SalFAD2*.*LIA*1 alleles *LIA* ^*1a*^, *LIA* ^*1b*^ and *lia* ^*1*^, and *SalFAD*2.*LIA*2 alleles *LIA* ^*2*^ and *lia* ^*2*^ in Yeast. Gas chromatography analysis of fatty acid composition of yeast cells containing the construct pYES2.1/V5-His-TOPO-LIA^1a^ with *LIA* ^1*a*^ coding DNA sequence (CDS), pYES2.1/V5-His-TOPO-LIA^1b^ with *LIA* ^*1b*^ CDS, pYES2.1/V5-His-TOPO-lia^1^ with *lia* ^*1*^ CDS, pYES2.1/V5-His-TOPO-LIA^2^ with *LIA* ^2^ CDS, pYES2.1/V5-His-TOPO-*lia* ^2^ with *lia* ^*2*^ CDS and the empty vector pYES2.1/V5-His-TOPO. **Statistical significant difference at *p* = 0.01 level.

Transgenic yeast cultures containing the empty construct pYES2.1/V5-His-TOPO produced the typical fatty acids, *i*.*e*. C16:0, C16:1, C18:0 and C18:1, found in untransformed cells (Fig. 3). Transgenic yeast cells harbouring the yeast expression vector pYES2.1/V5-His-TOPO- *LIA* ^1*a*^ carrying *LIA* ^1*a*^ coding sequence and pYES2.1/V5-His-TOPO-*LIA* ^*1b*^ (*lia* ^*1*^) with *LIA* ^*1b*^ (*lia* ^*1*^) coding sequence produced C16:2 and C18:2 fatty acids, and both C16:2 and C18:2 contents were not significantly different between cultures expressing *LIA* ^*1a*^ versus *LIA* ^*1b*^ (*lia* ^*1*^) (Fig. 3). This result indicated that the cloned alleles *LIA* ^*1a*^ and *LIA* ^*1b*^ (*lia* ^*1*^) encoded functional enzymes capable of desaturation of C16:1 to C16:2 and C18:1 to C18:2. Transgenic yeast cells harbouring the constructs pYES2.1/V5-His-TOPO-*LIA* ^2^ with *LIA* ^2^ coding sequence and pYES2.1/V5-His-TOPO-*lia* ^2^ with *lia* ^2^ coding sequence produced different amounts of C16:2 and C18:2 fatty acids. The C16:2 (6.5% ± 0.6% SD) and C18:2 (12.6% ± 0.8% SD) contents of yeast cells expressing *LIA* ^2^ were significantly higher than the C16:2 content (2.6% ± 0.2% SD) (t = 6.49, *p* < 0.01) and C18:2 content (7.8% ± 0.4% SD) (t = 8.79, *p* < 0.01) of the yeast cells expressing *lia* ^2^ (Fig. 3). These data suggested that the cloned *SalFAD*2.*LIA*2 alleles *LIA* ^2^ and *lia* ^2^ are functional, but the enzyme encoded by *lia* ^2^ produced lower C16:2 and C18:2 contents than that by *LIA* ^2^. The C16:2 (2.5% ± 0.04% SD) and C18:2 (9.7% ± 0.3% SD) contents of the yeast culture expressing *LIA* ^1*a*^ were significantly lower than the C16:2 content (6.5%) (t = 3.95, *p* < 0.01) and C18:2 content (12.6%) (t = 3.36, *p* < 0.01) of the yeast cells expressing *LIA* ^2^ (Fig. 3), implying that the enzyme encoded by *LIA* ^2^ produced higher C16:2 and C18:2 contents than that by *LIA* ^1*a*^. In summary, the cloned *SalFAD*2.*LIA1* alleles *LIA* ^*1a*^, *LIA* ^*1b*^ and *lia* ^*1*^ and the *SalFAD*2.*LIA2* alleles *LIA* ^*2*^ and *lia* ^*2*^ encoded functional palmitoleate and oleate desaturases that were capable of converting C16:1 to C16:2 and C18:1 to C18:2, but control different C16:2 and C18:2 contents.

### Transcription analysis of the *SalFAD2*.*LIA*1 and *SalFAD*2.*LIA*2 alleles

**Figure 4:**
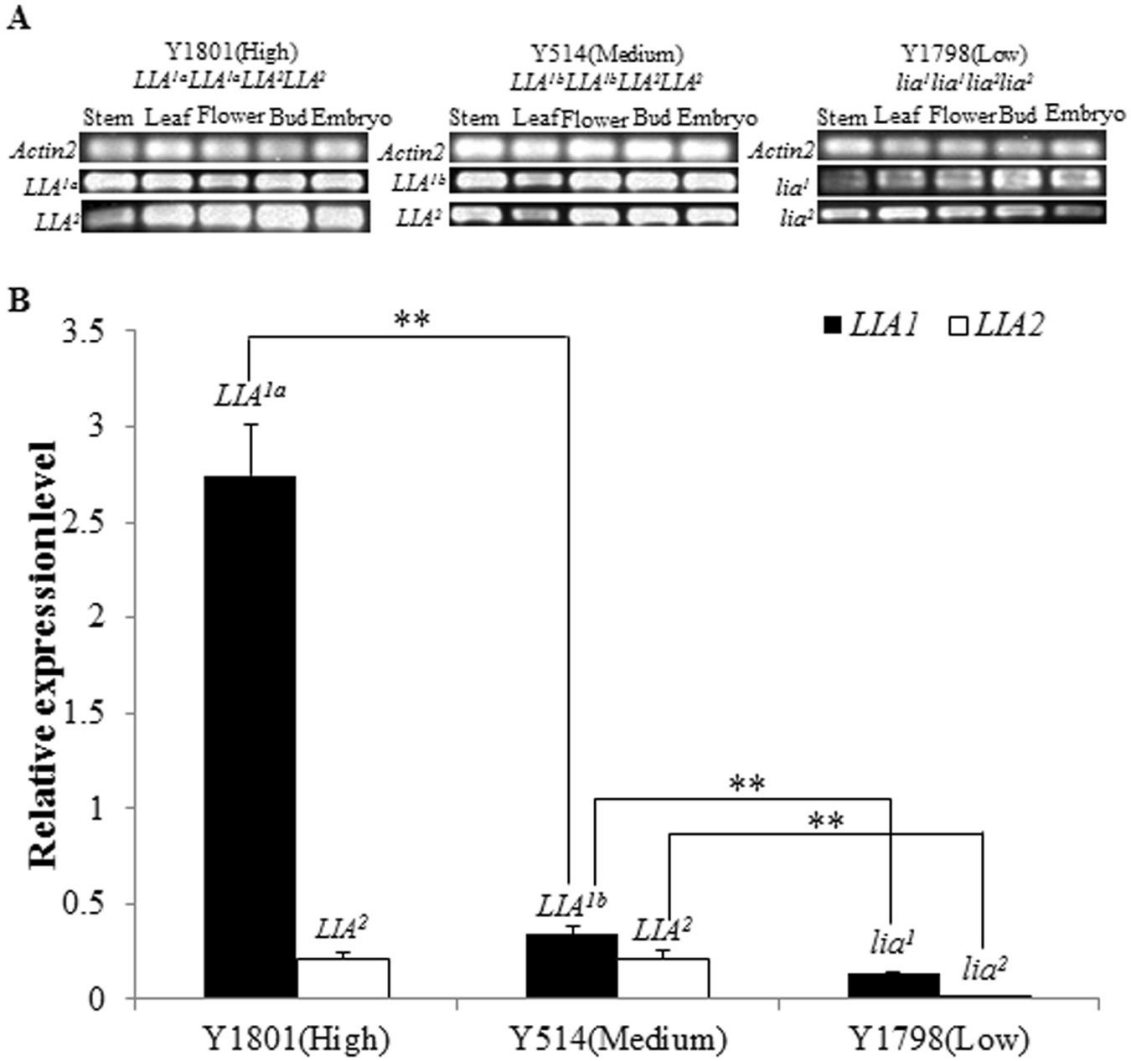
Expression analysis of the *SalFAD2*.*LIA*1 alleles *LIA* ^*1a*^, *LIA* ^*1b*^ and *lia* ^*1*^, and *SalFAD*2.*LIA*2 alleles *LIA* ^2^ and *lia* ^2^. Actin 2 (FG576123) was used as an internal control. A. *LIA* ^*1a*^, *LIA* ^*1b*^, *lia* ^*1*^, *LIA* ^2^ and *lia* ^2^ were expressed in the stem, leaf, flower, bud and 18d old embryo as revealed by RT-PCR. B. Quantitative Real-time PCR analysis of expression levels of the *LIA* ^*1a*^, *LIA* ^*1b*^, *lia* ^*1*^, *LIA* ^2^ and *lia* ^2^ in 18d- old embryos of Y1798 (low), Y514 (medium) and Y1801 (high). Error bars indicated SD of the mean. **Statistical significant difference at *p* = 0.01 level.

Transcripts of the *SalFAD2*.*LIA*1 and *SalFAD*2.*LIA*2 genes were detected in stem, leaf, flower bud and 18d-old embryo of Y1801, Y514 and Y1798 (Fig. 4A), suggesting that *LIA* ^1*a*^, *LIA* ^1*b*^, *lia* ^*1*^, *LIA* ^2^ and *lia* ^2^ were constitutively expressed in the plant. The RT-PCR bands from *LIA* ^*1a*^, *LIA* ^*1b*^, *lia* ^*1*^, *LIA* ^2^ and *lia* ^2^ (Fig. 4A) were cloned and sequenced, confirming the presence of a single copy. *LIA* ^1*a*^, *LIA* ^*1b*^, *lia* ^*1*^, *LIA* ^2^ and *lia* ^2^ exhibited differences in the transcription level in the 18d-old embryo. *LIA* ^*1a*^ in Y1801 had the highest transcription level (Fig. 4B), and the transcription levels of *LIA* ^*1b*^ in Y514 and *lia* ^*1*^ in Y1798 were 12.3% (SD 0.1%) and 5.0% (SD 0.1%) of that of *LIA* ^*1a*^, respectively. *lia* ^*1*^ had the lowest transcription level, at 40.8% (SD 0.1%) of *LIA* ^*1b*^. *LIA* ^2^ in Y1801 and *LIA* ^2^ in Y514 had the same transcription level. *lia* ^2^ in Y1798 had a very low transcription level, at 1.1% (SD 0.1%) of *LIA* ^2^ (Fig. 4B). The transcription levels of *LIA* ^*1a*^, *LIA* ^*1b*^, *lia* ^*1*^, *LIA* ^2^ and *lia* ^2^ were correlated with the C18:2 contents in the seeds of Y1801, Y514 and Y1798.

### Functional analysis of the putative promoters of the *SalFAD*2.*LIA1* and *SalFAD*2.*LIA*2 alleles

**Figure 5:**
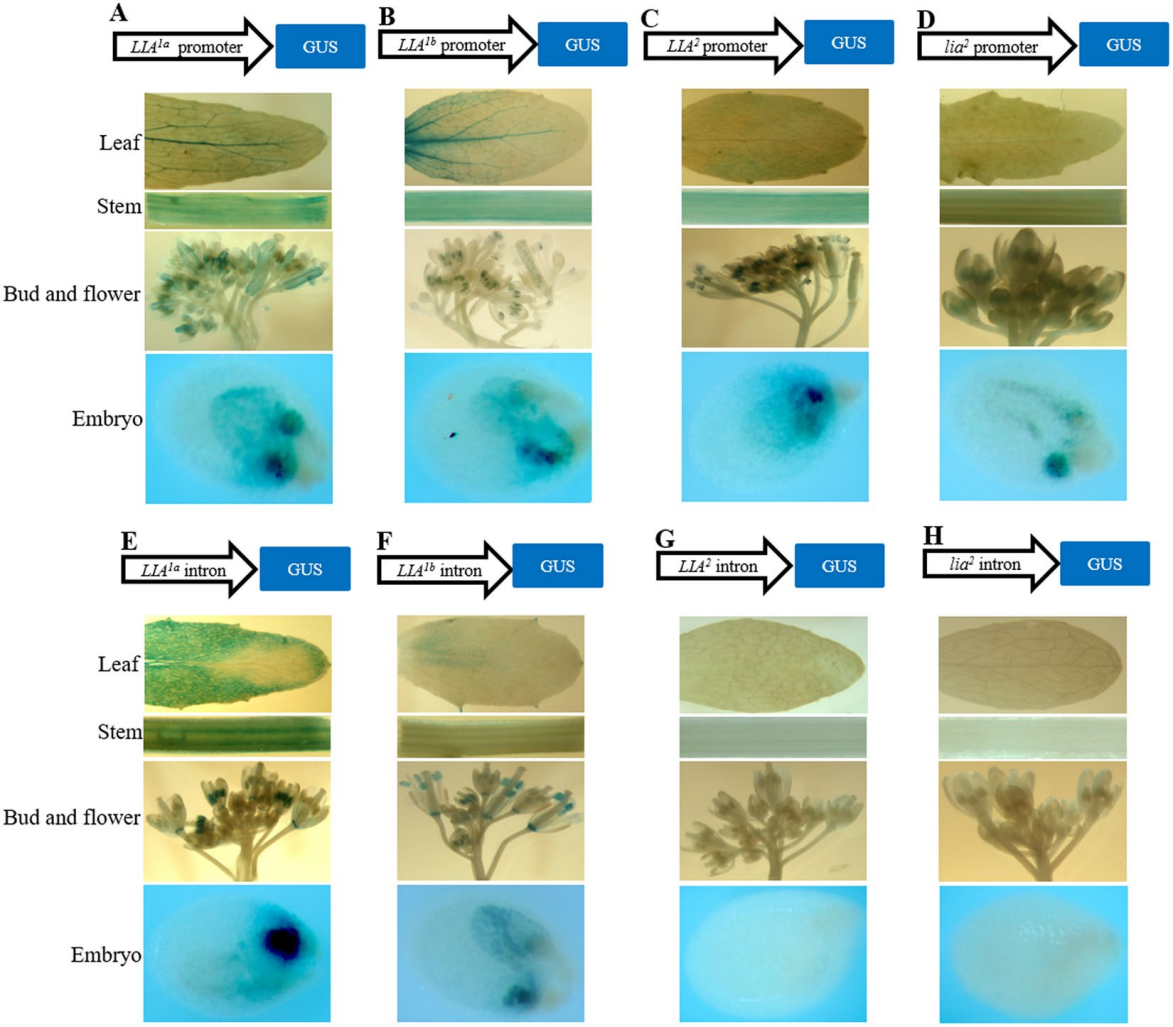
Histochemical localization of GUS activity in the leaf, stem, bud and flower, and 7d old embryo of transgenic *Arabidopsis* plants containing the construct pBI101-*LIA* ^*1a*^-promoter-GUS with the promoter of *LIA* ^*1a*^ (**A**), pBI101-*LIA* ^*1b*^-promoter-GUS with the promoter of *LIA* ^*1b*^ (**B**), pBI101-*LIA* ^2^-promoter-GUS with the promoter of *LIA* ^2^ (**C**), pBI101-*lia* ^2^-promoter-GUS with the promoter of *lia* ^2^ (**D**), pBI101-*LIA* ^1*a*^-intron-GUS with the intron of *LIA* ^1*a*^ (**E**), pBI101-*LIA* ^*1b*^-intron-GUS with the intron of *LIA* ^*1b*^ (**F**), pBI101-*LIA* ^2^-intron-GUS with the intron of *LIA* ^2^ (**G**) and pBI101-*lia* ^*2*^-intron-GUS with the intron of *lia* ^*2*^ (**H**), respectively. GUS staining was observed in the figures A, B, C, D, E and F, but not in the figures G and H.

The constructs pBI101-*LIA* ^*1a*^-promoter-GUS, pBI101-*LIA* ^*1b*^-promoter-GUS, pBI101-*LIA* ^2^-promoter-GUS and pBI101-*lia* ^2^ *-*promoter-GUS, carrying the putative promoter fragment of *LIA* ^*1a*^, *LIA* ^*1b*^, *LIA* ^2^ and *lia* ^2^, respectively, were transformed into *Arabidopsis*. Histochemical assays led to the detection of GUS activity in the leaf, stem, bud, flower tissues and embryo (Fig. 5A–D) in the transgenic plants containing the constructs pBI101-*LIA* ^*1a*^-Promoter-GUS, pBI101-*LIA* ^*1b*^-Promoter-GUS, pBI101-*LIA* ^2^-Promoter-GUS and pBI101-*lia* ^*2*^-Promoter-GUS. These results indicated the putative promoter fragments of *LIA* ^*1a*^, *LIA* ^*1b*^, *LIA* ^*2*^ and *lia* ^*2*^ functioned as a constitutive promoter. Based on the extent of GUS staining, the promoters of *LIA* ^*1a*^ and *LIA* ^*1b*^ appeared to have similar level of expression, while the promoter of *LIA* ^*2*^ had a higher activity than that of *lia* ^*2*^.

### The introns of the *SalFAD2*.*LIA1* alleles *LIA*^*1a*^ and *LIA*^*1b*^ function as a constitutive promoter

The constructs pBI101-*LIA* ^*1a*^-intron-GUS, pBI101-*LIA* ^*1b*^-intron-GUS, pBI101-*LIA* ^*2*^-intron-GUS and pBI101-*lia* ^*2*^-intron-GUS containing the intron fragment of *LIA* ^*1a*^, *LIA* ^*1b*^, *LIA* ^*2*^ and *lia* ^*2*^, respectively, were transformed into *Arabidopsis*. The intron of *lia* ^1^ was not tested for promoter function as it had a 1223 bp insertion at position −26. GUS activity was detected in the leaf, stem, bud, flower and the embryo of the transgenic plants containing the constructs pBI101-*LIA* ^*1a*^-intron-GUS (Fig. 5E), pBI101-*LIA* ^*1b*^-intron-GUS (Fig. 5F), indicating that the introns of *LIA* ^*1a*^ and *LIA* ^*1b*^ had the function of a constitutive promoter. However, the intron of *LIA* ^*1b*^ had a weaker promoter activity than that of *LIA* ^*1a*^ based on the extent of GUS staining. GUS activity was not detected in the transgenic plants containing the constructs of pBI101-*LIA* ^2^-intron-GUS (Fig. 5G) and pBI101-*lia* ^2^-intron-GUS (Fig. 5H), suggesting that the introns of *LIA* ^2^ and *lia* ^2^ had no promoter function.

### Co-segregation of the *SalFAD2*.*LIA*1 and *SalFAD*2.*LIA*2 allele-specific markers with C18:2 contents in the F_2_ populations

**Figure 6:**
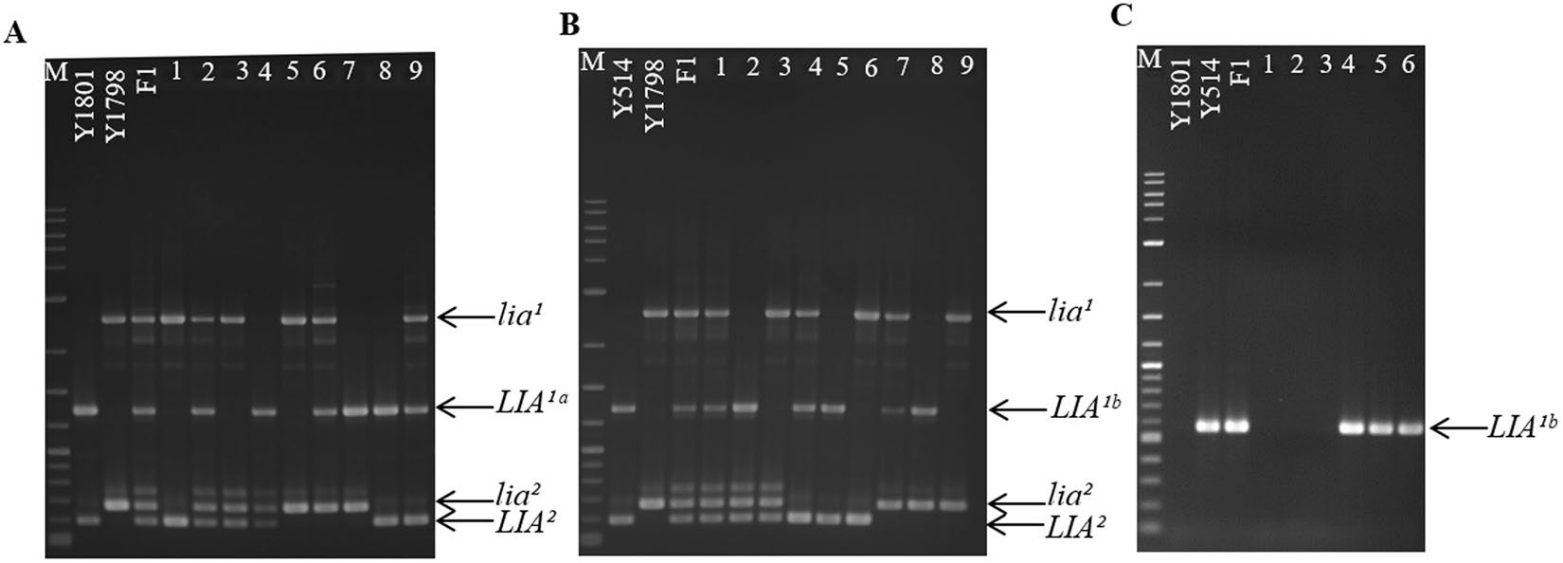
Identification of different C18:2 genotypes based on the *SalFAD2*.*LIA*1 and *SalFAD*2.*LIA*2 allele-specific markers in the F_2_ populations of the three crosses: (**A**) Y1798 (low) × Y1801 (high); B. Y1798 (low) × Y514 (medium) and C. Y1801 (high) × Y514 (medium). A. Y1801: high C18:2 line (*LIA* ^1*a*^ *LIA* ^1*a*^ *LIA* ^2^ *LIA* ^2^); Y1798: low C18:2 line (*lia* ^1^ *lia* ^*1*^ *lia* ^2^ *lia* ^2^); F_1_ (Y1798 × Y1801): *LIA* ^1*a*^ *lia* ^1^ *LIA* ^2^ *lia* ^2^; Lane 1: *lia* ^*1*^ *lia* ^*1*^ *LIA* ^2^ *LIA* ^2^; Lane 2: *LIA* ^1*a*^ *lia* ^1^ *LIA* ^2^ *lia* ^2^; Lane 3: *lia* ^1^ *lia* ^*1*^ *LIA* ^2^ *lia* ^2^. Lane 4: *LIA* ^*1a*^ *LIA* ^*1a*^ *LIA* ^2^ *lia* ^2^; Lane 5: *lia* ^*1*^ *lia* ^*1*^ *lia* ^2^ *lia* ^2^; Lane 6: *LIA* ^1*a*^ *lia* ^*1*^ *lia* ^2^ *lia* ^2^; Lane 7: *LIA* ^*1a*^ *LIA* ^*1a*^ *lia* ^2^ *lia* ^2^; Lane 8: *LIA* ^1*a*^ *LIA* ^1*a*^ *LIA* ^2^ *LIA* ^2^; Lane 9: *LIA* ^1*a*^ *lia* ^*1*^ *LIA* ^2^ *LIA* ^2^. (**B**) Y514: medium C18:2 line (*LIA* ^1*b*^ *LIA* ^*1b*^ *LIA* ^2^ *LIA* ^2^); Y1798: low C18:2 line (*lia* ^*1*^ *lia* ^*1*^ *lia* ^2^ *lia* ^2^); F_1_ (Y1798 × Y514): *LIA* ^1*b*^ *lia* ^1^ *LIA* ^2^ *lia* ^2^; Lane 1: *LIA* ^1*b*^ *lia* ^*1*^ *LIA* ^2^ *lia* ^2^; Lane 2: *LIA* ^*1b*^ *LIA* ^*1b*^ *LIA* ^2^ *lia* ^2^; Lane 3: *lia* ^1^ *lia* ^1^ *LIA* ^2^ *lia* ^2^. Lane 4: *LIA* ^1*b*^ *lia* ^*1*^ *LIA* ^2^ *LIA* ^2^; Lane 5: *LIA* ^*1b*^ *LIA* ^*1b*^ *LIA* ^2^ *LIA* ^2^; Lane 6: *lia* ^1^ *lia* ^1^ *LIA* ^2^ *LIA* ^2^; Lane 7: *LIA* ^*1b*^ *lia* ^*1*^ *lia* ^2^ *lia* ^2^; Lane 8: *LIA* ^*1b*^ *LIA* ^*1b*^ *lia* ^2^ *lia* ^2^; Lane 9: *lia* ^*1*^ *lia* ^*1*^ *lia* ^2^ *lia* ^2^. (**C**) Y1801: high C18:2 line (*LIA* ^*1a*^ *LIA* ^*1a*^ *LIA* ^2^ *LIA* ^2^); Y514: medium C18:2 line (*LIA* ^*1b*^ *LIA* ^*1b*^ *LIA* ^2^ *LIA* ^2^); F_1_ (Y514 × Y1801): *LIA* ^*1a*^ *LIA* ^*1b*^ *LIA* ^*2*^ *LIA* ^*2*^. 1–3: *LIA* ^*1a*^ *LIA* ^*1a*^ *LIA* ^*2*^ *LIA* ^*2*^; 4–6: -*LIA* ^*1b*^ *LIA* ^*2*^ *LIA* ^*2*^.

All of the nine possible genotypes were identified using the markers specific for *LIA* ^*1a*^, *lia* ^*1*^, *LIA* ^2^ and *lia* ^2^ in the cross of Y1798 (low) × Y1801 (high) (Fig. 6A). The homozygous F_2_ plants of *LIA* ^1*a*^ *LIA* ^*1a*^ *LIA* ^2^ *LIA* ^2^ had a significantly higher C18:2 content (average: 21.2%) than the heterozygous F_2_ plants of *LIA* ^*1a*^ *lia* ^*1*^ *LIA* ^2^ *LIA* ^2^ (average: 16.7%) (t = 3.08, *p* < 0.01) and that of *LIA* ^1*a*^ *LIA* ^*1a*^ *LIA* ^2^ *lia* ^2^ (average: 17.9%) (t = 2.68, *p* < 0.01). This result suggested that the allelic effect on the increase of C18:2 was *LIA* ^*1a*^ > *lia* ^*1*^ and *LIA* ^2^ > *lia* ^2^. The homozygous F_2_ plants of *LIA* ^1*a*^ *LIA* ^1*a*^ *lia* ^2^ *lia* ^2^ had a higher average C18:2 content (17.9%) than those of *lia* ^*1*^ *lia* ^*1*^ *LIA* ^2^ *LIA* ^*2*^ (average: 9.4%) (t = 7.80, *p* < 0.01), indicating that *LIA* ^1*a*^ controlled a higher C18:2 content than *LIA* ^2^. In the cross of Y1798 (low) × Y514 (medium), the nine genotypes for C18:2 content were distinguished from each other by the markers specific for *LIA* ^*1b*^, *lia* ^*1*^, *LIA* ^*2*^ and *lia* ^2^ (Fig. 6B). The average C18:2 content of the homozygous F_2_ plants (*LIA* ^*1b*^ *LIA* ^*1b*^ *LIA* ^2^ *LIA* ^2^) was 11.5%, which was significantly higher than that (average: 7.9%) of the heterozygous F_2_ plants of *LIA* ^1*b*^ *lia* ^*1*^ *LIA* ^2^ *LIA* ^2^ (t = 3.95, *p* < 0.01), implying that *LIA* ^1*b*^ contributed to a higher C18:2 content than *lia* ^*1*^.

In the cross of Y1801 (high) × Y514 (medium), the dominant marker specific for *LIA* ^*1b*^ was used to identify the F_2_ plants of *LIA* ^*1a*^ *LIA* ^*1a*^ *LIA* ^2^ *LIA* ^2^ from those of *LIA* ^*1a*^ *LIA* ^*1b*^ *LIA* ^2^ *LIA* ^2^ and *LIA* ^*1b*^ *LIA* ^*1b*^ *LIA* ^2^ *LIA* ^2^ (Fig. 6C). The homozygous F_2_ plants of *LIA* ^*1a*^ *LIA* ^*1a*^ *LIA* ^2^ *LIA* ^2^ had an average C18:2 content of 23.7%, which was higher than the bulked F_2_ plants of *LIA* ^*1a*^ *LIA* ^*1b*^ *LIA* ^2^ *LIA* ^2^ and *LIA* ^*1b*^ *LIA* ^*1b*^ *LIA* ^2^ *LIA* ^2^ (average: 21.9%) (t = 2.20, *p* = 0.02), suggesting that the allelic effect in increasing C18:2 content is *LIA* ^1*a*^ > *LIA* ^*1b*^. The *SalFAD*2.*LIA1* and *SalFAD*2.*LIA2* genes co-localized with the QTL peaks on Sal01 and Sal08, respectively (Fig. 1).

## Discussion

In the present study, QTL mapping revealed the occurrence of four C18:2 QTLs in yellow mustard. The C18:2 QTLs on Sal01 and Sal08 were responsible for 14.9–47.3% and 9.5% of the phenotypic variation and co-localized with the *FAD2* genes *SalFAD2*.*LIA*1 and *SalFAD*2.*LIA*2, respectively. However, the C18:2 QTL on Sal02 was mapped to the same region as the C18:3 QTL and *FAD3* gene *SALFAD3*.*LA1*, and the C18:2 QTL on Sal03 as the C22:1 QTL and the *FAE1* gene. The co-localization of the C18:2 QTL with the C18:3 and C22:1 QTLs could be due to the fact that the three fatty acids share the same biosynthetic pathway. Both C18:2 and C22:1 use C18:1n-9 as a starting substrate. The use of this substrate for C22:1 synthesis may result in less C18:1n-9 being available for C18:2 productions. Further desaturation of C18:2 by the *FAD3* gene leads to the production of C18:3 and the reduction of the C18:2 content.

The *SalFAD*2.*LIA1* and *SalFAD*2.*LIA*2 genes each contained two exons, one intron within the 5′-UTR and a promoter adjacent to the first exon, which is in agreement with that in *Arabidopsis* ^4^, *B*. *napus* ^5, 6, 32^ and other species including *G*. *hirstum* ^7^ and *V*. *labrusca* ^10^. The promoters and introns of *SalFAD*2.*LIA1* gene in yellow mustard demonstrated constitutive promoter activity as that of the *FAD*2 in *B*. *napus* ^6^. The protein encoded by each of the *SalFAD*2.*LIA1* alleles *LIA* ^1*a*^, *LIA* ^*1b*^ and *lia* ^*1*^ and *SalFAD*2.*LIA*2 alleles *LIA* ^2^ and *lia* ^*2*^ has six transmemberane domains and three H boxes harboring eight iron-binding histidines, which are consistent with previous reports in *Arabidopsis* ^4, 12^. The positions of the H boxes and histidines in the *FAD2* polypeptides in yellow mustard are in agreement with that in *Arabidopsis* (Supplementary Fig. S5). Heterologous expression in yeast confirmed that the cloned *SalFAD2*.*LIA*1 alleles *LIA* ^1*a*^ and *LIA* ^1*b*^(*lia* ^1^) and the *SalFAD2*.*LIA2* alleles *LIA* ^*2*^and *lia* ^*2*^ encoded functional palmitoleate and oleate desaturases that were capable of converting C16:1 to C16:2 and C18:1 to C18:2.

It has been reported that SNP/indel mutations in the coding region of the *FAD2* resulted in loss-of-function alleles in *B*. *napus* ^14–16^ and *B*. *rapa* ^17^. However, the mutations observed in the coding region of *SalFAD2*.*LIA*1 and *SalFAD*2.*LIA*2 genes in yellow mustard were different from those reported in *Brassica* species^14–17^. Moreover, this report reveals that extensive SNP and indel mutations in the intron and promoter as well as transposable element insertion in the intron are involved in modulating the expression of *FAD*2 in yellow mustard. The dominant *SalFAD*2.*LIA1* allele *LIA* ^1*a*^ conditioned a higher C18:2 content and had a promoter with stronger activity than the dominant *SalFAD2*.*LIA2* allele *LIA* ^*2*^. In addition, the intron of *LIA* ^*1a*^ demonstrated strong promoter activity, whereas that of *LIA* ^*2*^ lost the promoter function. Sequence alignment with *LIA* ^*1a*^ indicated that *LIA* ^*2*^ harboured 103 SNPs and 29 indels in the promoter, and 106 SNPs and 44 indels in the intron. Search for cis-regulatory elements in the promoters and introns of *LIA* ^*1a*^ and *LIA* ^*2*^ were performed using PlantCARE (http://bioinformatics.psb.ugent.be/webtools/plantcare/html/)^33^. The promoter of *LIA* ^*1a*^ contained 14 “CAAT box”, a common cis-acting element in the promoter and enhancer regions; while that of *LIA* ^*2*^ had only 5 “CAAT box”. The reduction of the number of “CAAT box” might account for the reduced promoter activity of *LIA* ^*2*^. The intron of *LIA* ^*1a*^ contained 24 “CAAT box”. However, the intron of *LIA* ^2^ had only 9 “CAAT box”. Moreover, based on the prediction of possible transcription promoter using BDGP (http://www.fruitfly.org/seq_tools/promoter.html)^34^, the intron of *LIA* ^*1a*^ contained the predicted transcription starting site “T” at nucleotide 825 and putative “TATA-box” TTTAAAA at position 787 (Supplementary Fig. S12). However, the intron of *LIA* ^2^ did not have the possible transcription promoter, which might have led to the loss of the promoter function. Therefore, it could be inferred that the mutations in the promoter and intron of *LIA* ^2^ resulted in reduced transcription level and C18:2 content.

The *SalFAD*2.*LIA1* allele *LIA* ^*1b*^ differed from *LIA* ^*1a*^ in the CDS, promoter and intron, and controlled a lower C18:2 content than *LIA* ^*1a*^. Expression studies in *S*. *cerevisiae* indicated that *LIA* ^*1a*^ and *LIA* ^*1b*^ produced similar levels of C16:2 and C18:2 fatty acids although the proteins encoded by *LIA* ^*1b*^ harboured the amino acid changes at positions 72 and 84. Sequence alignment with *LIA* ^*1a*^ indicated that the promoter of *LIA* ^*1b*^ carried 9 SNP and 8 indel mutations. However, based on GUS staining, the promoters of *LIA* ^*1a*^ and *LIA* ^*1b*^ appeared to have similar level of expression. Therefore, the mutations of the CDS and promoter of *LIA* ^*1b*^ had no effect on the biosynthesis of C18:2. Sixteen indels and 49 SNP mutations occurred in the intron of *LIA* ^*1b*^ compared with that of *LIA* ^*1a*^. Searching for cis-regulatory elements indicated that the intron of *LIA* ^*1a*^ contains the motif “5UTR Py-rich stretch” (TTTCTTCTTT) (nucleotides 856–866) (Supplementary Fig. S9) which can enhance transcription level. However, the intron of *LIA* ^*1b*^ had lost this motif due to point mutations at positions 868, 871 and 872 (Supplementary Fig. S9). This might lead to the reduction of the promoter activity of the intron in *LIA* ^*1b*^. Thus, it could be concluded that the mutation in the intron of *LIA* ^*1b*^ resulted in the reduction of C18:2 content. The recessive *SalFAD*2.*LIA1* allele *lia* ^*1*^ produced a lower C18:2 content than *LIA* ^*1b*^. The allele *lia* ^*1*^ was identical to *LIA* ^*1b*^ in the promoter, exon 1 and exon 2, but harboured a 1223 bp transposable element insertion at position −26 bp in the 5′ upstream region from the translation start codon. The insertion of the retrotransposon in the intron of *LIA* ^*1b*^ might have generated the allele *lia* ^*1*^ with further reduced transcription level and C18:2 content. There are two possible explanations for this: Firstly, the inserted retrotransposon of *lia* ^*1*^ might disturb the interaction of the promoter, intron and other transcriptional factors, thereby affecting *FAD*2 expression. Secondly, the promoter of *lia* ^*1*^ is further away from the CDS due to the insertion, which might result in a reduction in transcription efficiency compared to *LIA* ^*1b*^, where the regulatory region is adjacent to the coding region. Insertions of DNA transposons or retrotransposons within or near genes have been reported to negatively affect the expression of genes by decreasing or abolishing transcription^35–39^.

The recessive *SalFAD*2.*LIA2* allele *lia* ^*2*^ controlled a lower C18:2 content than *LIA* ^*2*^, which could be due to the mutations of the CDS and promoter since the introns of both *LIA* ^*2*^ and *lia* ^*2*^ lost the promoter function. Yeast cells expressing *lia* ^2^ had significantly lower C16:2 and C18:2 contents than the yeast cells expressing *LIA* ^2^. Compared with *LIA* ^*2*^, the polypeptide encoded by *lia* ^*2*^ comprised six amino acid changes at positions 12, 42, 58, 63, 169 and 206 (Supplementary Fig. S5), which might result in reduced enzyme activity. GUS staining indicated that the promoter of *lia* ^*2*^ was weaker and had a lower expression level than that of *LIA* ^*2*^. Sequence alignment with *LIA* ^*2*^ revealed that the promoter of *lia* ^2^ harboured 9 indels. The predicted site of “TATA-box” in the promoter region of *lia* ^2^ was different from that of *LIA* ^2^ due to a 10 bp insertion occurred at position 368 (Supplementary Fig. S11). This might have resulted in the reduced promoter function of *lia* ^*2*^. Therefore, it was concluded that *lia* ^*2*^ produced a low C18:2 content due to the mutations of both CDS and promoter.

In conclusion, this study revealed that the linoleic acid variation was controlled by multiple alleles with different expression levels at two gene loci, *SalFAD2*.*LIA*1 and *SalFAD*2.*LIA2*, in yellow mustard. The results demonstrated that complex quantitative genetic variation of trait phenotype could be caused by multiple alleles of oligogenic loci resulting from mutations in regulatory regions such as promoter and intron as well as CDS.

## Materials and Methods

### Plant materials

Y1798 and Y1801, developed via pedigree breeding from the variety Andante, are S_4_ and S_5_ inbred lines, respectively. Y514 is the doubled haploid line SaMD3^30^. Linoleic, linolenic (C18:3) and erucic (C22:1) acid contents of the three parental lines Y1798, Y514 and Y1801 are shown in Table 1. Y1798 has a low C18:2 content (average: 4.2%; range: 3.5–5.3%), an average C18:3 content of 13.0% (range: 10.9–15.5%) and an average C22:1 content of 36.3% (range: 11.2–45.6%). Y514 has a medium C18:2 content (average: 12.5%; range: 12.0–13.0%), an average C18:3 content of 10.8% (range: 9.6–12.4%) and zero C22:1 content. Y1801 has a high C18:2 content (average: 31.5%; range: 28.7–37.7%), an average C18:3 content of 12.5% (range: 10.2–14.5%) and an average C22:1 content of 16.1% (range: 14.7–17.3%). The C18:3 and C22:1 genotypes of Y1798, Y514 and Y1801 were determined using the two *FAD3* genes, *SalFAD3*.*LA*1 and *SalFAD3*.*LA*2, and *FAE1* gene allele-specific markers^27, 40^ (Supplementary Table S3). Y1798 carried the *FAE1* allele *E* ^*1*^ for a high C22:1 content, and *SalFAD3*.*LA1* allele *LA* ^*1a*^ and *SalFAD3*.*LA*2 allele *LA* ^*2*^ for a high C18:3 content. Y514 had the *FAE*1 allele *e* for zero C22:1 content, and *SalFAD3*.*LA1* allele *LA* ^*1*^ for a high C18:3 content and *SalFA3*.*LA*2 allele *la* ^*2*^ for a low C18:3 content. Y1801 harboured the *FAE*1 allele *E* ^2^ for a medium C22:1 content and the *SalFAD3*.*LA1* allele *la* ^*1*^ for a low C18:3 content and *SalFA3*.*LA*2 allele *LA* ^*2*^ for a high C18:3 content (Supplementary Table S3). The F_1_ seeds of the three crosses Y1798 (low) × Y1801 (high), Y1798 (low) × Y514 (medium) and Y1801 (high) × Y514 (medium) were produced. The F_1_ plants were self-pollinated to produce F_2_ seeds. One hundred and twenty-three F_2_ plants from one F_1_ plants of Y1798 × Y1801, 122 F_2_ plants from one F_1_ plant of Y1798 × Y514 and 122 F_2_ plants from one F_1_ plant of Y1801 × Y514 were used for constructing the genetic linkage map and QTL mapping of C18:2, C18:3 and C22:1 contents. All plants were raised under the same conditions in the greenhouse at Agriculture and Agri-Food Canada-Saskatoon Research and Development Centre.

### Seed fatty acid analysis

Seed fatty acid composition was analyzed according to Thies (1971)^41^ with the following modification: the gas chromatography of the methyl esters was performed with a HP-INNOWax fused silica capillary column (0.25 mm by 0.5 m and 7.5 µm) (Agilent Technologies) at 250 °C using hydrogen as the carrier gas. A minimum of 10 seeds from each of the parental lines and F_1_ hybrids, and 150 F_2_ seeds of each of the crosses Y1798 × Y1801, Y1798 × Y514 and Y1801 × Y514 were half-seed analyzed according to Downey and Harvey (1963)^42^.

### Cloning of the coding regions of the *SalFAD2*.*LIA*1 alleles *LIA*^*1a*^, *LIA*^*1b*^ and *lia*^*1*^, and *SalFAD*2.*LIA*2 alleles *LIA*^*2*^ and *lia*^*2*^

Primer pair No 1 (Supplementary Table S1) was designed based on the conserved coding regions of *BnFAD2* (FJ907397) from *B*. *napus* and *AtFAD2* (TAIR: At3G12120) from *A*. *thaliana*, and used to clone the CDS of the *FAD2* alleles *LIA* ^1*a*^, *LIA* ^*1b*^, *lia* ^*1*^, *LIA* ^2^ and *lia* ^2^ in yellow mustard. Genomic DNA of each of the three parental lines Y1798, Y514 and Y1801 was used as template for PCR amplification with fideli-Taq DNA polymerase (Affymetrix) in a thermocycler with 30 cycles of the following program: 94 °C for 30 s, 56 °C for 30 s, and 68 °C for 2 min.

### Cloning the 3′ and 5′ flanking sequences of the coding regions of the *SalFAD2*.*LIA*1 alleles *LIA*^*1a*^, *LIA*^*1b*^and *lia*^*1*^, and *SalFAD*2.*LIA*2 alleles *LIA*^*2*^ and *lia*^*2*^

The primer pairs used to clone the 3′ and 5′ flanking sequences of the coding regions of the *SalFAD2*.*LIA*1 alleles *LIA* ^*1a*^, *LIA* ^*1b*^ and *lia* ^*1*^, and *SalFAD*2.*LIA*2 alleles *LIA* ^*2*^ and *lia* ^*2*^ are presented in Supplementary Table S1 and Supplementary Fig. S4. Primer pair No 2 was designed based on the sequence of the 3′ coding region of the *FAD2* alleles *LIA* ^1*a*^, *LIA* ^1*b*^, *lia* ^1^, *LIA* ^*2*^ and *lia* ^*2*^ and used to clone the 3′ downstream sequence of each of the five alleles. Primer pair No 3, designed based on the 5′ coding region of *LIA* ^1*a*^, *LIA* ^*1b*^ and *lia* ^*1*^, was used to clone the 5′ upstream sequences of the three alleles. Primer pair No 4 were designed based on the 5′ upstream sequence obtained from the first round of PCR walking to clone the further upstream sequences of *LIA* ^*1a*^, *LIA* ^*1b*^ and *lia* ^*1*^. Primer pair No 5, designed based on the promoter sequence of *BnFAD*2 (KF038144), was used to clone the promoter region of *LIA* ^*1a*^, *LIA* ^*1b*^ and *lia* ^*1*^. Primer pair No 6 was designed to clone the DNA fragment between the promoter and 5′ flanking sequence of *LIA* ^*1a*^, *LIA* ^*1b*^ and *lia* ^*1*^. Primer pair No 7, designed based on the 5′ coding region was used to clone the 5′ upstream sequences of *LIA* ^2^ and *lia* ^2^. Primer pair No 8, designed based on the 5′ upstream sequence obtained from the first round of PCR walking to clone the further upstream sequences of *LIA* ^2^ and *lia* ^*2*^. PCR walking was performed according to the protocol of Siebert *et al*. (1995)^43^. The standard protocol from the clontech kit (Protocol PT 3042, Version PR 03300) was followed to facilitate the PCR walking. Primer pair No 9 was designed to amplify *LIA* ^1*a*^, *LIA* ^*1b*^ and *lia* ^*1*^ and primer pair No 10 was designed to amplify *LIA* ^2^ and *lia* ^*2*^ in their entirety. The sequence analysis of the inserted DNA fragment in the intron of the *SalFAD2*.*LIA*1 allele *lia* ^1^ was according to Zeng and Cheng (2014)^27^. Primer pair No 11 (Supplementary Table S1) was designed based on the exon 1 (nucleotide: 1 to 20) and exon 2 (nucleotide: 1156–1137) of *LIA* ^1a^ to obtain the cDNA sequence of the *SalFAD*2.*LIA1* alleles *LIA* ^*1a*^, *LIA* ^*1b*^, *lia* ^*1*^. Primer pair No 12 (Supplementary Table S1) was designed based on the exon 1 (nucleotide: 1 to 20) and exon 2 (nucleotide: 1162–1143) of *LIA* ^2^ to get the cDNA sequence of the *SalFAD*2.*LIA2* alleles *LIA* ^*2*^ and *lia* ^*2*^.

### Transformation of yeast and fatty acid analysis

The coding sequences of the *SalFAD2*.*LIA*1 alleles *LIA* ^*1a*^ and *LIA* ^*1b*^ (*lia* ^*1*^) were amplified using primer pair No 13 and those of the *SalFAD*2.*LIA*2 alleles *LIA* ^*2*^ and *lia* ^*2*^ using primer pair No 14 (Supplementary Table S1). The amplified sequences of *LIA* ^1*a*^, *LIA* ^*1b*^ (*lia* ^*1*^), *LIA* ^2^ and *lia* ^2^ were then cloned into the pYES2.1/V5-His-TOPO expression vector (Invitrogen), respectively, and sequenced to confirm the correct orientation of genes. The four constructs pYES2.1/V5-His-TOPO-*LIA* ^1*a*^, pYES2.1/V5-His-TOPO-*LIA* ^*1b*^ (*lia* ^*1*^), pYES2.1/V5-His-TOPO-*LIA* ^2^ and pYES2.1/V5-His-TOPO-*lia* ^2^ were transformed into *Saccharomyces cerevisiae* strain Inv Sc1 (Invitrogen), by the lithium acetate method^44^. Yeast cells transformed with the empty vector pYES2.1/V5-His-TOPO plasmid were used as a control. Transgenic cells were screened in complete minimal drop-out uracil medium containing 2% raffinose as a carbon source at 30 °C. The positive clones were grown at 30 °C overnight in minimal media supplemented with 2% raffinose and lacking uracil. The transgenic cell culture was centrifuged, followed by washing, and then used to inoculate 20 ml induction media (minimal media lacking uracil and supplemented with 2% galactose and 1% raffinose) to an OD_600_ of 0.5. Cultures were grown overnight. The fatty acid composition analysis was the same as previously reported^27^.

### RT-PCR and Quantitative Real-time PCR

Total RNA was extracted from leaf, stem, flower bud, flower and 18-d old embryos of Y1798, Y514 and Y1801 using an RNeasy plant mini kit (Qiagen). Extracted RNA was treated with DNase I (Ambion), and cDNA was synthesized using the ReverTra Ace-a-First Strand cDNA synthesis kit (Thermo Fisher) according to the manufacturer’s instruction.

Primer pair No 15 (Supplementary Table S1) was designed based on exon 1 and exon 2 sequences of the *SalFAD2*.*LIA*1 alleles *LIA* ^*1a*^, *LIA* ^*1b*^ and *lia* ^*1*^, while primer pair No 16 (Supplementary Table S1) was designed based on exon 1 and exon 2 sequences of the *SalFAD*2.*LIA*2 alleles of *LIA* ^*2*^ and *lia* ^*2*^. To further detect the differences in transcription level, quantitative PCR (qPCR) analysis was conducted on 18d old embryos of Y1798, Y514 and Y1801. Primer pair No 17 (Supplementary Table S1), which was used to amplify the 155 bp fragment (nucleotides 11 to 165) of the cDNA, specific for the *SalFAD2*.*LIA*1 alleles *LIA* ^*1a*^, *LIA* ^*1b*^ and *lia* ^*1*^. Primer pair No 18 (Supplementary Table S1), which was used to amplify the 191 bp fragments (nucleotides 106 to 296) of the cDNA, specific for the *SalFAD*2.*LIA*2 alleles *LIA* ^2^ and *lia* ^2^. The qPCR analysis was performed with SsoFast EvaGreen supermix (Bio-Rad) according to the manufacturer’s instructions using a Bio-Rad CFX96^TM^ system. Primer pair No 19 (Supplementary Table S1) specific for Actin2 (FG576123) was used as an internal control for normalization. Three separate first strand cDNA reactions were analyzed in duplicate for each sample, and expression levels were calculated as described by Livak and Schmittgen (2001)^45^.

### Transformation of *Arabidopsis* and histochemical GUS Assays

The putative promoter sequences of 639 bp, 714 bp, 566 bp and 554 bp for *LIA* ^*1a*^, *LIA* ^*1b*^, *LIA* ^2^ and *lia* ^*2*^ were cloned using primer pairs No 20, 21, 22 and 23, respectively (Supplementary Table S1), and inserted into the plant expression vector pBI101 upstream of the GUS gene. The intron sequences of 1286 bp, 1354 bp, 579 bp and 617 bp for *LIA* ^*1a*^, *LIA* ^*1b*^, *LIA* ^*2*^ and *lia* ^2^ were cloned using primer pairs No 24, 25, 26 and 27, respectively (Supplementary Table S1) and were also inserted into the plant expression vector pBI101 upstream of the GUS gene. The resulting constructs were transformed into electrocompetent *Agrobacterium* cells GV3101 by electroporation according to the manufacturer’s instructions. *Agrobacterium*-mediated transformation of wild-type *Arabidopsis* plants was performed according to Clough and Bent (1998)^46^. Transgenic plants were screened and analyzed according to Jako *et al*.^47^. For each construct, at least 10 positive plants were used for analysis. Embryos of 7d-old, leaf, stem, bud and flower tissues of the transgenic plants were stained overnight in solution containing 0.5 mg/ml 5-bromo-4-chloro-3-indolyl-beta-D-glucuronide, 50 mM Na phosphate buffer, pH 7.0, 1 mM K3Fe(CN)6, 1 mM K4Fe(CN)6, 20 mM EDTA. The staining of the embryo and vegetative tissues was observed and photographed using a Zeiss stereomicroscope with a color CCD camera.

### Development of the *SalFAD2*.*LIA*1 and *SalFAD*2.*LIA*2 allele-specific markers

Primer pair No 28 (Supplementary Table S1), which was designed based on the conserved flanking sequences of the intron of the *SalFAD2*.*LIA*1 and *SalFAD*2.*LIA*2 genes and produced co-dominant markers of 1317 bp, 1329 bp, 2549 bp, 607 bp and 682 bp specific for the *SalFAD2*.*LIA*1 alleles *LIA* ^*1a*^, *LIA* ^*1b*^ and *lia* ^*1*^, and *SalFAD*2.*LIA*2 alleles *LIA* ^*2*^ and *lia* ^*2*^, respectively. The PCR was performed using phusion high-fidelity polymerase (NEB) with 30 cycles of the following program: 98 °C for 10 s, 60 °C for 30 s, and 72 °C for 2 min. Primer pair No 29 (Supplementary Table S1), designed based on the 12 bp insertion in the promoter region of *LIA* ^1*b*^, generating a dominant marker of 548 bp specific for *LIA* ^*1b*^. The PCR amplification was the following program of 30 cycles: 94 °C for 30 s, 56 °C for 30 s, and 72 °C for 1 min.

### Construction of a linkage map and QTL analysis

A total of 785 ILP primer pairs, 164 from *A*. *thaliana*, 316 from *B*. *napus* and 305 from *B*. *rapa*, were used to screen the parental lines Y1798 (low), Y514 (medium) and Y1801 (high) for polymorphic markers. Of the 785 primer pairs, 130, 110 and 114 polymorphic markers were generated between the parental lines Y1798 and Y1801, Y1798 and Y514, and Y1801 and Y514, respectively. The genetic linkage map was constructed by using JoinMap^48^ version 4.0 at LOD scores ≥ 4.0. Chi-square test for goodness-of-fit was performed to determine if marker segregation deviated from the expected ratio. The threshold of *p* < 0.01 was used to exclude the distorted markers from the map construction. An MQM mapping analysis was conducted using the MapQTL 6.0 software^49^ to detect QTLs for C18:2, C18:3 and C22:1 contents. Permutation test (1,000 replications) was used to determine the significance level for LOD with a genome-wide probability of *p* < 0.05. QTL analysis was performed for C18:2, linolenic (C18:3) and erucic (C22:1) contents in the F_2_ populations derived from the three crosses Y1798 (low) × Y1801 (high), Y1798 (low) × Y514 (medium) and Y1801 (high) × Y514 (medium). The effects of the *SalFAD*2.*LIA1* alleles *LIA* ^*1a*^, *LIA* ^*1b*^ and *lia* ^*1*^, and *SalFAD*2.*LIA2* alleles *LIA* ^*2*^ and *lia* ^*2*^ on linoleic (C18:2) acid content was estimated by ANOVA procedure.

### Accession numbers

The GenBank accession numbers for the nucleotide sequences of the *SalFAD2*.*LIA*1 alleles *LIA* ^*1a*^, *LIA* ^*1b*^ and *lia* ^*1*^ are KY305533, KY305534, and KY305535 and *SalFAD*2.*LIA*2 alleles *LIA* ^*2*^ and *lia* ^*2*^ are KY305536 and KY305537, respectively.

## Electronic supplementary material

Supplemental Data
