# Supplementary material for: Mutations in the promoter, intron and CDS of two FAD2 generate multiple alleles modulating linoleic acid level in yellow mustard
Source: Sci Rep. 2017 Aug 15;7:8284. doi: 10.1038/s41598-017-08317-y (PMC5557838; doi:10.1038/s41598-017-08317-y)
Supplement: Supplementary file 1 — Supplemental Data [file 41598_2017_8317_MOESM1_ESM.pdf]

The following Supporting Information is available for this article

Supplemental Figure 1 Boxplot of linoleic acid content of lines Y1798 (low), Y514 (medium), Y1801 (high), middle parent (MP) value, and F_1 and F_2 seeds of Y1798 (low) × Y1801(high) (A); Y1798 (low) × Y514 (medium) (B) and Y1801 (high) × Y514 (medium) (C).

Supplemental Figure 2 Frequency distributions of linoleic acid content in individual seeds of the three F_2 populations Y1798 (low) × Y1801(high) (A), Y1798 (low) × Y514 (medium) (B) and Y1801(high) × Y514 (medium)(C).

Supplemental Figure 3A Linkage map constructed using the F_2 population derived from the cross of Y1798 (low) × Y1801 (high). Chromosome assignment was based on Javidfar and Cheng 2013. C18:2, C18:3 and C22:1 QTLs are marked in black, green and red colours respectively. 1-LOD and 2-LOD supporting intervals of each QTL are marked with thick and thin bars, respectively.

Supplemental Figure 3B Linkage map constructed using the F_2 population derived from the cross of Y1798 (low) × Y514 (medium). Chromosome assignment was based on Javidfar and Cheng 2013. C18:2, C18:3 and C22:1 QTLs are marked in black, green and red colours respectively. 1-LOD and 2-LOD supporting intervals of each QTL are marked with thick and thin bars, respectively.

Supplemental Figure 3C Linkage map constructed using the F_2 population derived from the cross of Y1801 (high) × Y514 (medium). Chromosome assignment was based on Javidfar and Cheng 2013. C18:2, C18:3 and C22:1 QTLs are marked in black, green and red colours respectively. 1-LOD and 2-LOD supporting intervals of each QTL are marked with thick and thin bars, respectively.

Supplemental Figure 4 Schematic diagram showing the position of each primer used for cloning of the Sal*FAD2.LIA1* alleles LIA^{1a}, LIA^{1b}, lia^{1}, and *SalFAD2.LIA2* alleles LIA^{2} and lia^{2}.

Supplemental Figure 5 Alignment of amino acid sequences encoded by the *SalFAD2.LIA1* alleles LIA^{1a}, LIA^{1b} (lia^{1}) and *SalFAD2.LIA2* alleles LIA^{2} and lia^{2} of yellow mustard.

Supplemental Figure 6 PCR amplification of the entire *SalFAD2.LIA1* alleles LIA^{1a}, LIA^{1b}, lia^{1} (A) and *SalFAD2.LIA2* alleles LIA^{2} and lia^{2} (B) using the primer pair No 9 and 10, respectively (Table S1).

Supplemental Figure 7 Sequence alignment of the introns of LIA^{1b} and lia^{1}.

Supplemental Figure 8 Sequence alignment of the putative promoters (A) and exon 1 (B) of the *SalFAD2.LIA1* alleles *LIA*^{1a}, *LIA*^{1b} and *lia*^{1} in yellow mustard.

Supplemental Figure 9 Sequence alignment of the introns of the *SalFAD2.LIA1* alleles *LIA*^{1a} and *LIA*^{1b}.

Supplemental Figure 10 Sequences analysis of the 1223 bp insertion happped in the intron of *lia*^{1}.

Supplemental Figure 11 Sequence alignments of the promoters, exon 1 and introns of the *SalFAD2.LIA2* alleles *LIA*^{2} and *lia*^{2}.

Supplemental Figure 12 Sequence alignments of the putative promoters and introns of the dominant *SalFAD2.LIA1* allele *LIA*^{1a} and the *SalFAD2.LIA2* allele *LIA*^{2}.

Supplemental Table S**1** Primer pairs used in this study

Supplemental Table S**2** Summary of QTLs for C18:2, C18:3 and C22:1 contents in the F_2 populations derived from the three crosses Y1798 (low) × Y1801 (high), Y1798 (low) × Y514 (medium) and Y1801 (high) × Y514 (medium)

Supplemental Table S3 The C18:3 and C22:1 genotypes of the parental lines Y1798, Y514 and Y1801 were determined using the *FAD3* genes, *SalFAD3.LA1* and *SalFAD3.LA2*, and *FAE1* gene allele-specific markers, respectively.

Supplemental Figure 1 Boxplot of linoleic acid content of lines Y1798 (low), Y514 (medium), Y1801 (high), middle parent (MP) value, and F_1 and F_2 seeds of Y1798 (low) × Y1801(high) (A); Y1798 (low) × Y514 (medium) (B) and Y1801 (high) × Y514 (medium) (C).

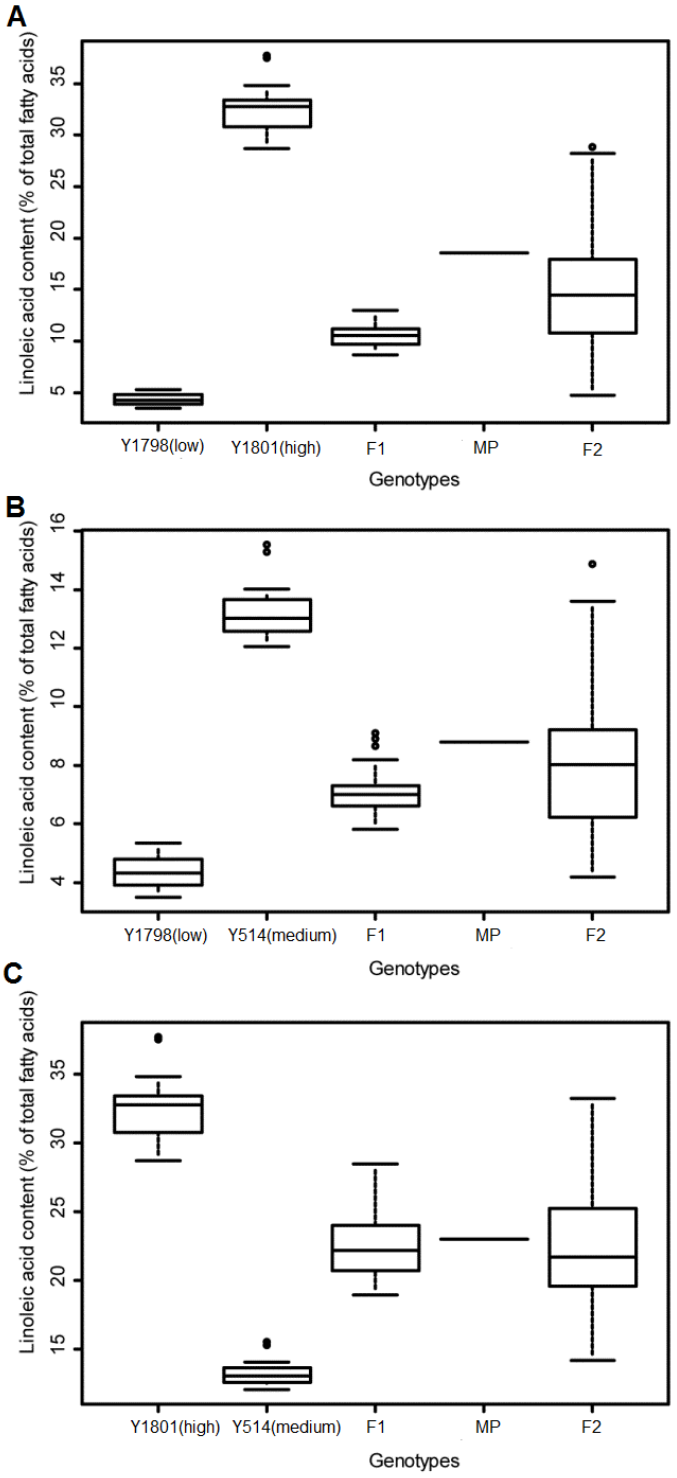

Supplemental Figure 2 Frequency distributions of linoleic acid content in individual seeds of the three F_2 populations Y1798 (low) × Y1801 (high) (A), Y1798 (low)× Y514 (medium) (B) and Y1801 (high) × Y514 (medium)(C).

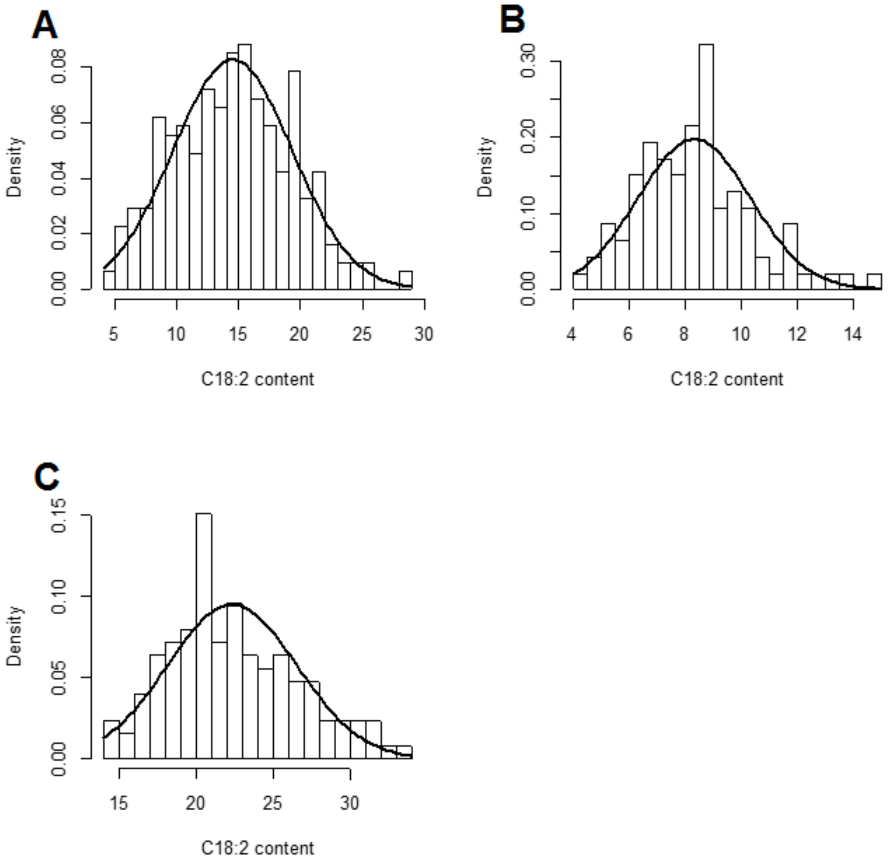

Supplemental Figure 3A Linkage map constructed using the F_2 population derived from the cross of Y1798 (low) × Y1801 (high). Chromosome assignment was based on Javidfar and Cheng 2013. C18:2 , C18:3 and C22:1 QTLs are marked in black, green and red colours respectively. 1-LOD and 2-LOD supporting intervals of each QTL are marked with thick and thin bars, respectively.

A

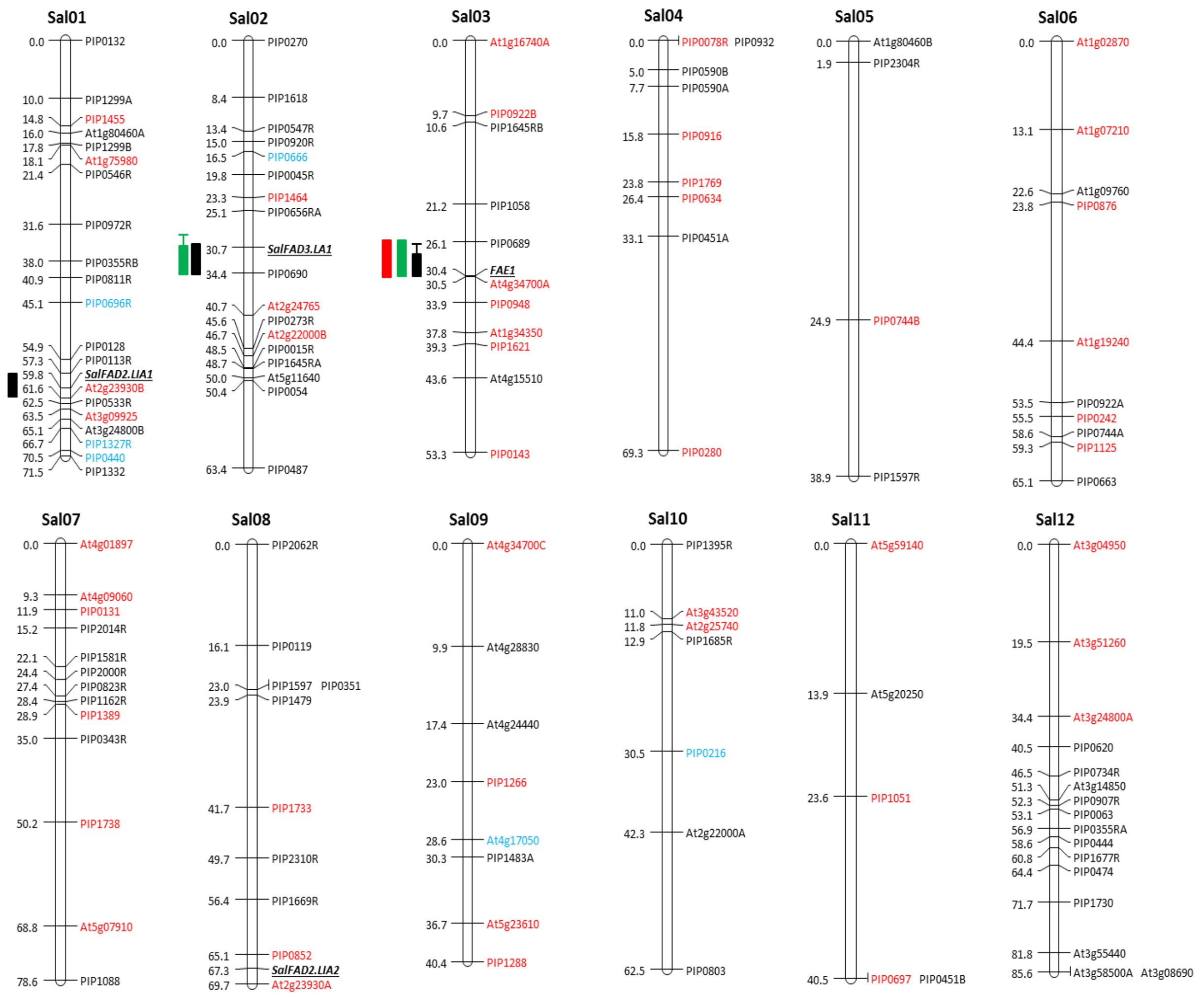

Supplemental Figure 3B Linkage map constructed using the F_2 population derived from the cross of Y1798 (low) × Y514 (medium). Chromosome assignment was based on Javidfar and Cheng 2013. C18:2 , C18:3 and C22:1 QTLs are marked in black, green and red colours respectively. 1-LOD and 2-LOD supporting intervals of each QTL are marked with thick and thin bars, respectively.

B

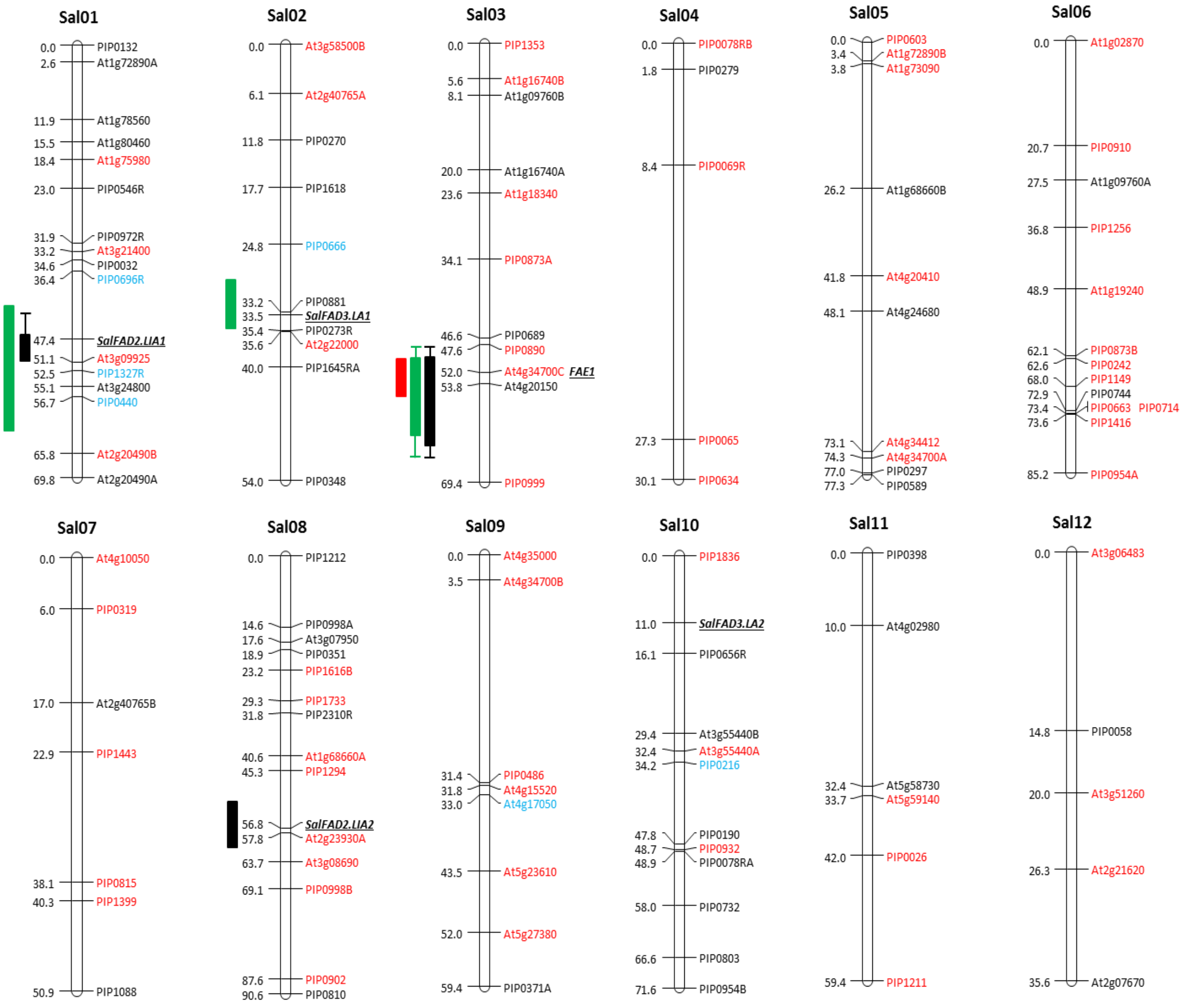

Supplemental Figure 3C Linkage map constructed using the F_2 population derived from the cross of Y1801 (high) × Y514 (medium). Chromosome assignment was based on Javidfar and Cheng 2013. C18:2 , C18:3 and C22:1 QTLs are marked in black, green and red colours respectively. 1-LOD and 2-LOD supporting intervals of each QTL are marked with thick and thin bars, respectively.

C

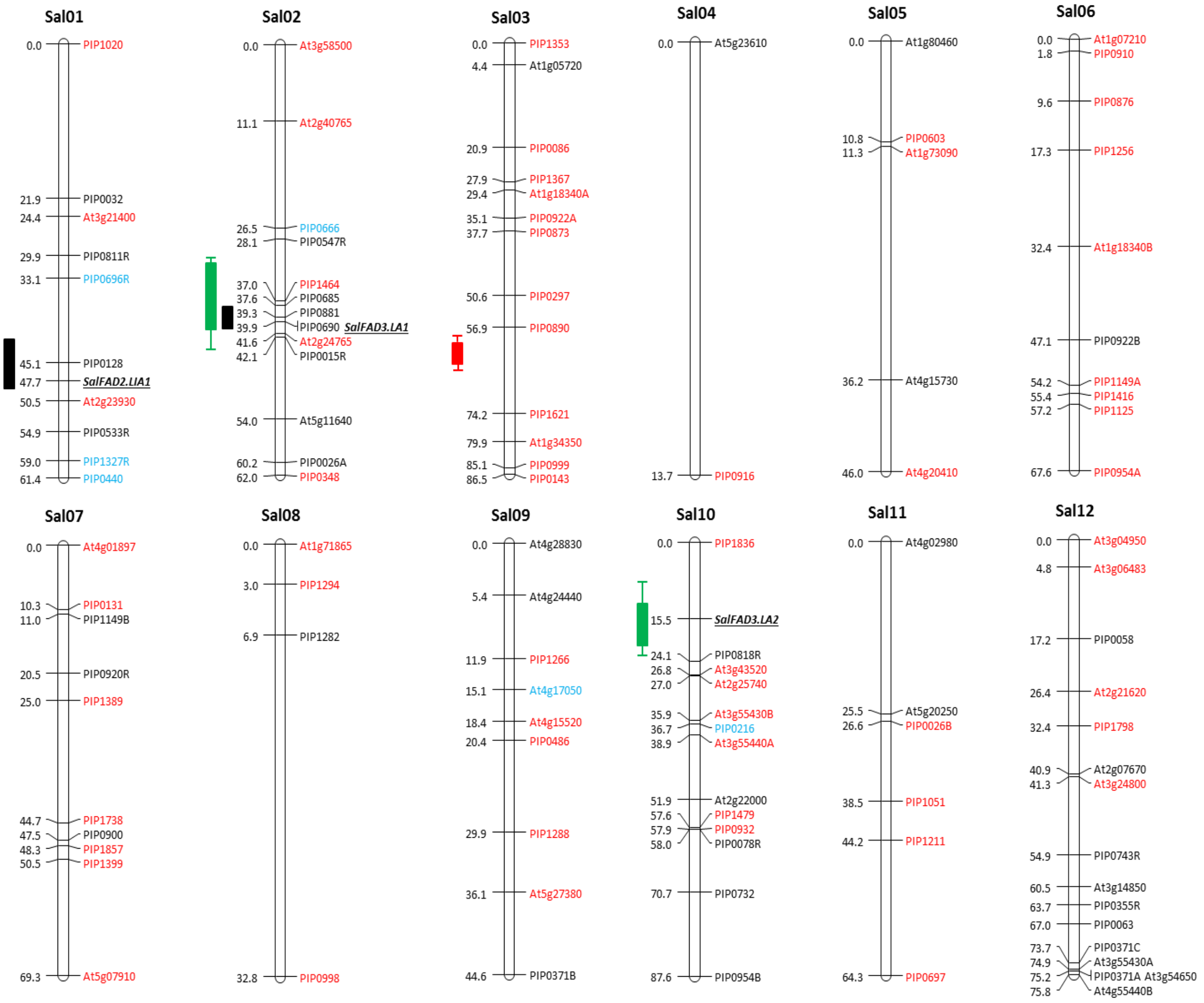

Supplemental Figure 4 Schematic diagram showing the position of each primer used for cloning of the Sal*FAD2.LIA1* alleles LIA^{1a}, LIA^{1b}, lia^{1}, and *SalFAD2.LIA2* alleles LIA^{2} and lia^{2}. Arrows indicated the direction of the primers. Black rectangle: Exon; Straight line: Intron; Grey rectangle: 768 bp 3' UTR; Solid black arrow: Promoter.

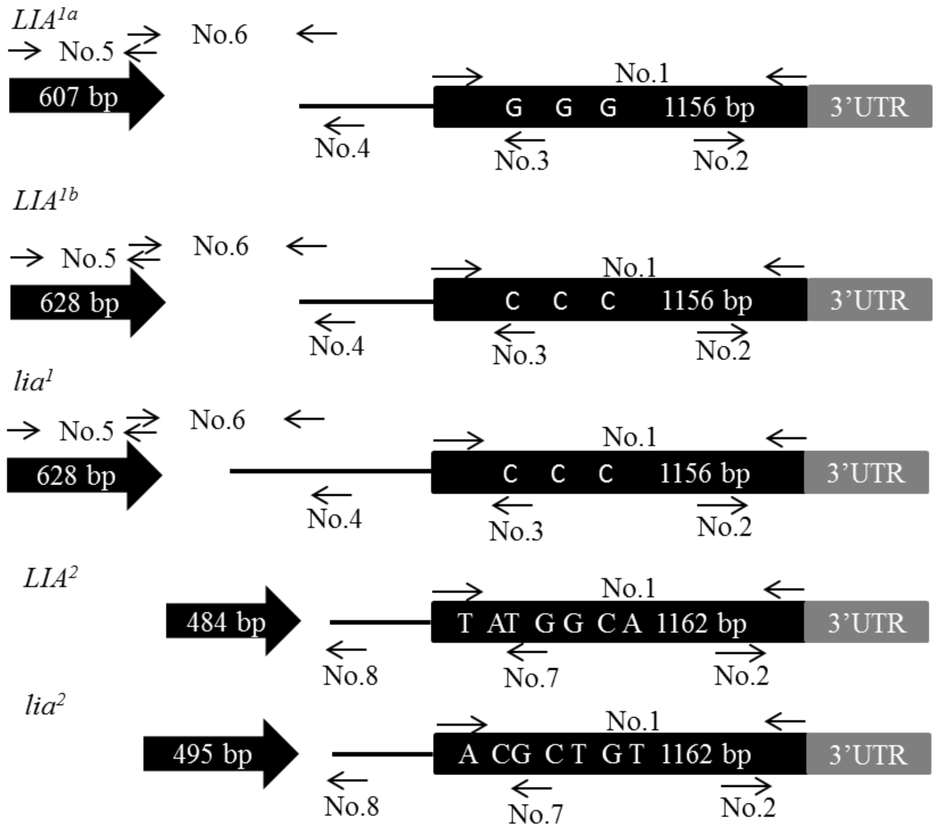

Supplemental Figure 5 Alignment of amino acid sequences encoded by the *SalFAD2.LIA1* alleles *LIA*^{1a}, *LIA*^{1b} (*lia*^{1}) and *SalFAD2.LIA2* alleles *LIA*^{2} and *lia*^{2} of yellow mustard. The amino acid sequence alignment was carried out using the ClustalW program (http://www.ebi.ac.uk/Tools/msa/clustalw2/). Six transmembrane domains and three histidine boxes are indicated by red box and line, respectively. Blue diamonds indicate the amino acid changes between the proteins encoded by *LIA*^{1a} and *LIA*^{1b} (*lia*^{1}) at poisitons 72 and 84. Red triangles show the six amino acid changes between the proteins encoded by *LIA*^{2} and *lia*^{2}. The green stars indicate the two amino acid lost at positions 175 and 205 in the protein encoded by *LIA*^{1a} compared with that encoded by *LIA*^{2}. Black inverted triangles indicate 27 amino acid changes between the proteins encoded by *LIA*^{1a} and *LIA*^{2}.

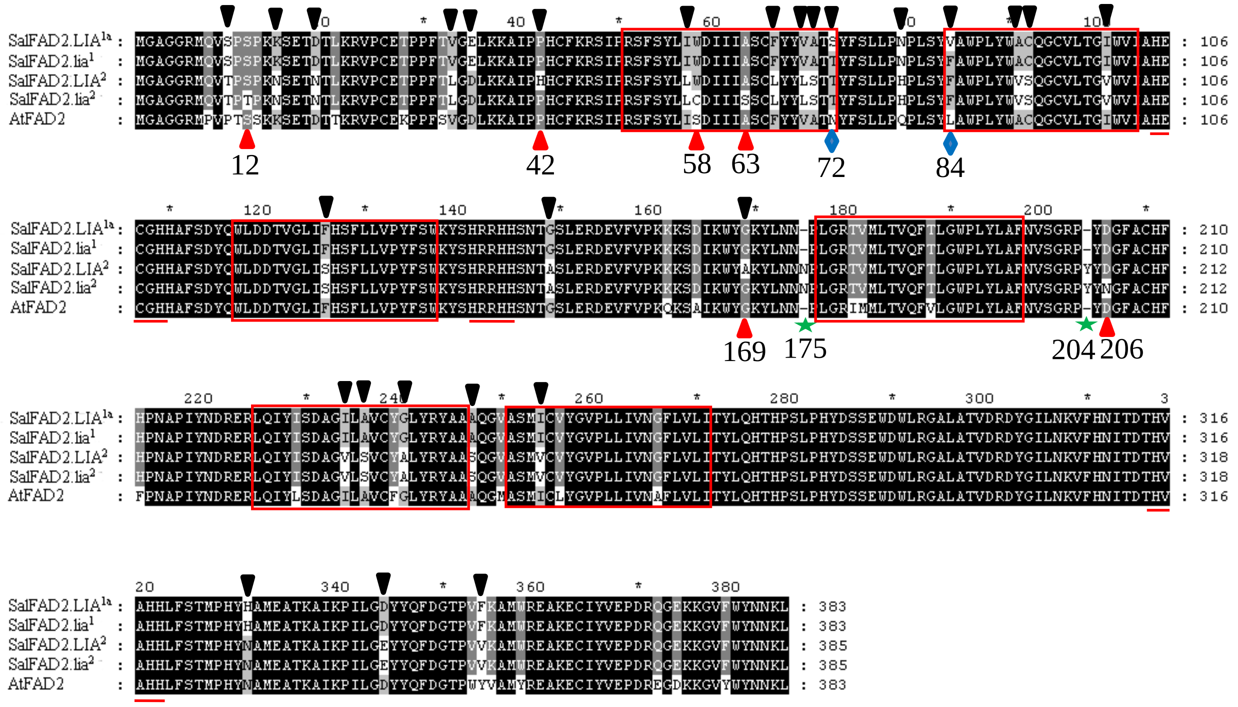

Supplemental Figure 6 PCR amplification of the entire *SalFAD2.LIA1* alleles LIA^{1a}, LIA^{1b}, lia^{1} (A) and *SalFAD2.LIA2* alleles LIA^{2} and lia^{2} (B) using the primer pair No 9 and 10, respectively (Table S1).

A. Lane 1: 1 kb DNA ladder; Lanes 2 and 3: 3150 bp fragment of LIA^{1a} in Y1801(high); Lanes 4 and 5: 3181 bp fragment of LIA^{1b} in Y514 (medium); Lanes 6 and 7: 4404 bp fragment of lia^{1} in Y1798 (low).

B. Lane 1: 1 kb DNA ladder; Lanes 2 and 3: 2744 bp fragment of lia^{2} in Y1798 (low); Lanes 4 and 5: 2281 bp fragment of LIA^{2} in Y514 (medium); Lanes 6 and 7: 2281 bp fragment of LIA^{2} in Y1801 (high).

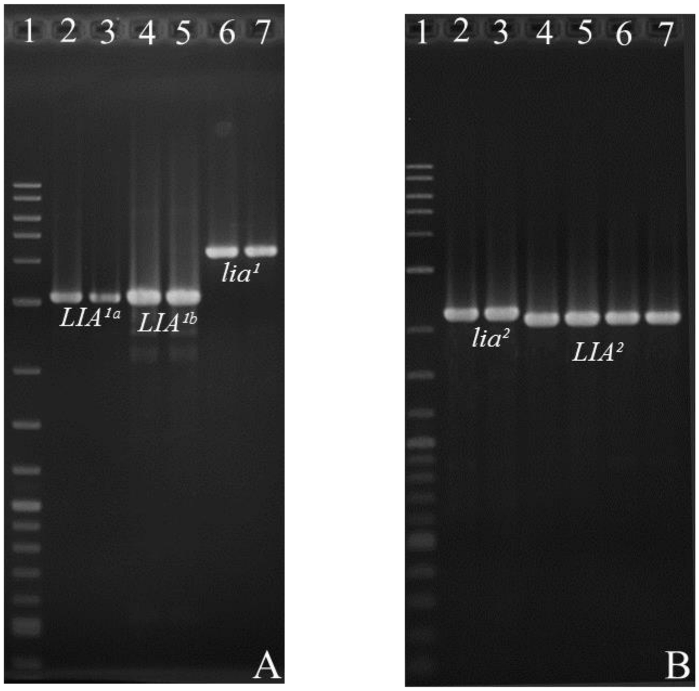

Supplemental Figure 7 Sequence alignment of the intron regions of *LIA*^{1b} and *lia*^{1}.

Red inverted triangle indicates the insertion of 1223 bp at position -26 in the intron sequence of *lia*^{1}. The A of start codon ‘ATG’ was designated as ‘1’.

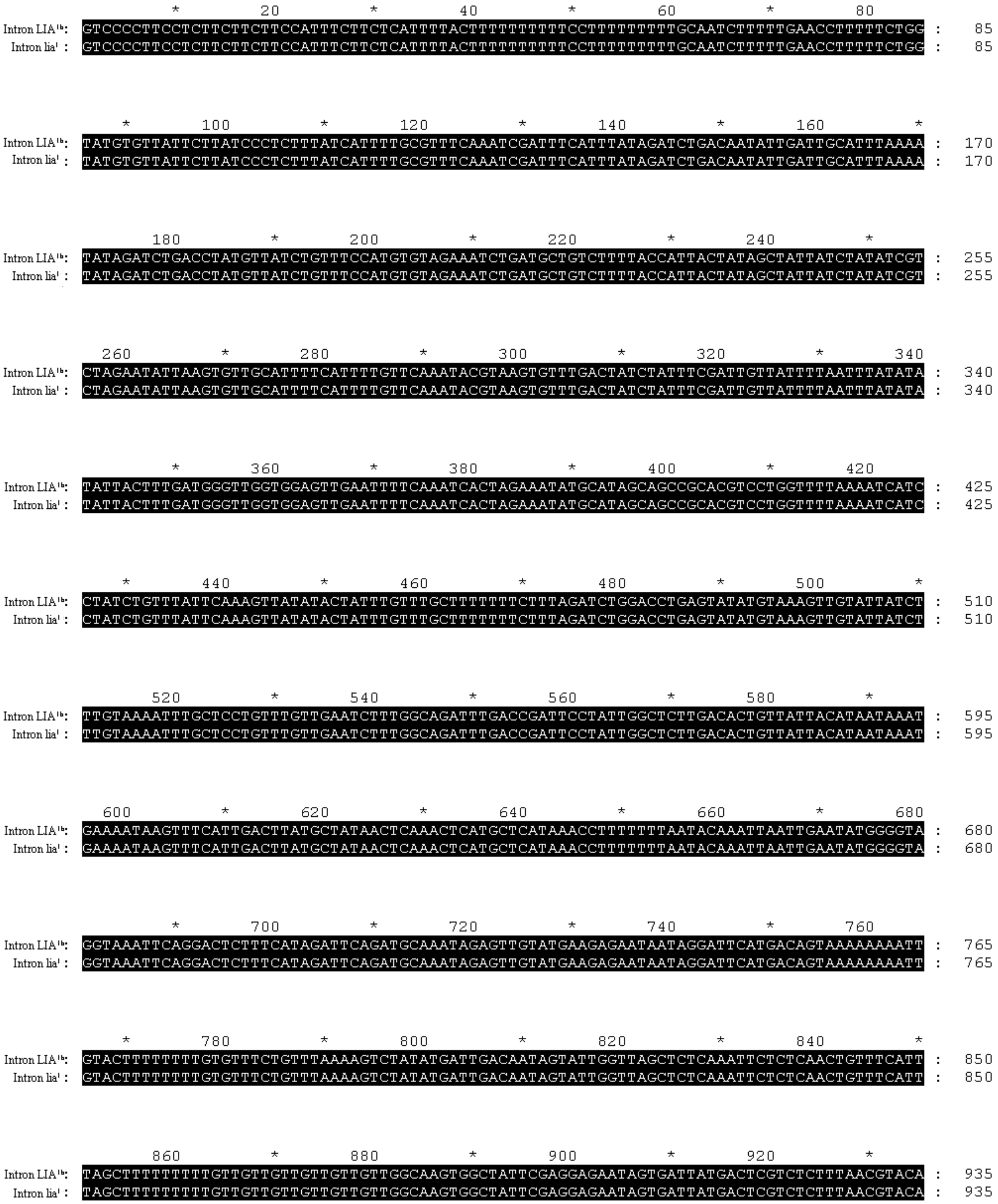

940 * 960 * 980 * 1000 * 1020
Intron LIA^{lb}: TTAGTAAAAGATAAAGAAATTGATATCCACAAGAAAGAGATGTGAGCTGTGTCGTATCAAATCTCATTAATTTACTTGTAGTATT : 1020
Intron lia^{l} : TTAGTAAAAGATAAAGAAATTGATATCCACAAGAAAGAGATGTGAGCTGTGTCGTATCAAATCTCATTAATTTACTTGTAGTATT : 1020

133

* 1040 * 1060 * 1080 * 1100
Intron LIA^{lb}: CTCAACGCTACCGTTTCTTTCTTTCTTTGGTTTGCTACTAAATGCCGATTCCTCTCTCTTTGTTCCTTTTGTTCCACGTACTATC : 1105
Intron lia^{l} : CTCAACGCTACCGTTTCTTTCTTTCTTTGGTTTGCTACTAAATGCCGATTCCTCTCTCTTTGTTCCTTTTGTTCCACGTACTATC : 1105

134

* 1120 * 1140 * 1160 * 1180 *
Intron LIA^{lb}: CATTTTTGTGGTTATCCACTTTTTAAAAAAACTTAACTATGAATCTTTTGTCTTAAGAATAATATGTTGGTTTAGTCGTTAGCTT : 1190
Intron lia^{l} : CATTTTTGTGGTTATCCACTTTTTAAAAAAACTTAACTATGAATCTTTTGTCTTAAGAATAATATGTTGGTTTAGTCGTTAGCTT : 1190

135

1200 * 1220 * 1240 * 1260 *
Intron LIA^{lb}: TGGATATTGAGTCTT-- : 1205
Intron lia^{l} : TGGATATTGAGTCTTATTAGGTGTATAGTATTGTATACTTGTATATGAGTAAGTTAGAGTAGAGTAATACACACATGTATATATA : 1275

136

1280 * 1300 * 1320 * 1340 * 1360
Intron LIA^{lb}: --- : -
Intron lia^{l} : GTGATCATTGTAACAACCTTTAATCTTAATGAAGATATTTCCATTTCATCGTCTTTCTGTTATGGTATCAGAGCGCATGAGCTGA : 1360

137

* 1380 * 1400 * 1420 * 1440
Intron LIA^{lb}: --- : -
Intron lia^{l} : GCTCAGCGATTTCTCCGATCCGTTCTCCGATCATCGTCTTCATTCTTCCGATCATCATCTTCTTCATACCGTTCTTCGATCATCA : 1445

138

* 1460 * 1480 * 1500 * 1520 *
Intron LIA^{lb}: --- : -
Intron lia^{l} : TCTCCGTTCTCCGATCATCATCTTCTGTTCACTTTCCTCCTCGGGTTCCTCATCATCTCAAGTCTTCCGCGCCATCTCGATTCCG : 1530

139

1540 * 1560 * 1580 * 1600 *
Intron LIA^{lb}: --- : -
Intron lia^{l} : TTTTATTTCTGATTTCATTTGATTCCCGATCTTCATCATGGGGAAGAGAAAAGTGTAATTTCGAAGTACAGAAGCTCAACTCCCC : 1615

140

1620 * 1640 * 1660 * 1680 * 1700
Intron LIA^{lb}: --- : -
Intron lia^{l} : AAGAAGCTTCAATCTCCGACTCATCGCGAATCGTCTCCAACCTCCGATCCTCGTCGCGACGCATCCGGGATCTTCACATCAGGCT : 1700

141

* 1720 * 1740 * 1760 * 1780
Intron LIA^{lb}: --- : -
Intron lia^{l} : GTTCGTCTTCCTAATCTTGATAATCCAGTTGCTATTCGTTCTACTAAGCCGTTTGAGGTATCCAACTCTCCAGATAACATACATA : 1785

142

* 1800 * 1820 * 1840 * 1860 *
Intron LIA^{lb}: --- : -
Intron lia^{l} : GTCCCATATTTTCTACATAGTTCGGATCATCCAGGCTTAGTGCTCGCATCTGAGGCATTAGATGGAATGAACTATAGTATCTGGA : 1870

143

1880 * 1900 * 1920 * 1940 *
Intron LIA^{lb}: --- : -
Intron lia^{l} : CCATTGCTATGACTACTAGCTTAGAGGCGAAGAACAAGCTTGGTTTTATTGATGGATCTATTGTTATGCCGGCTGAAACTGATCC : 1955

144

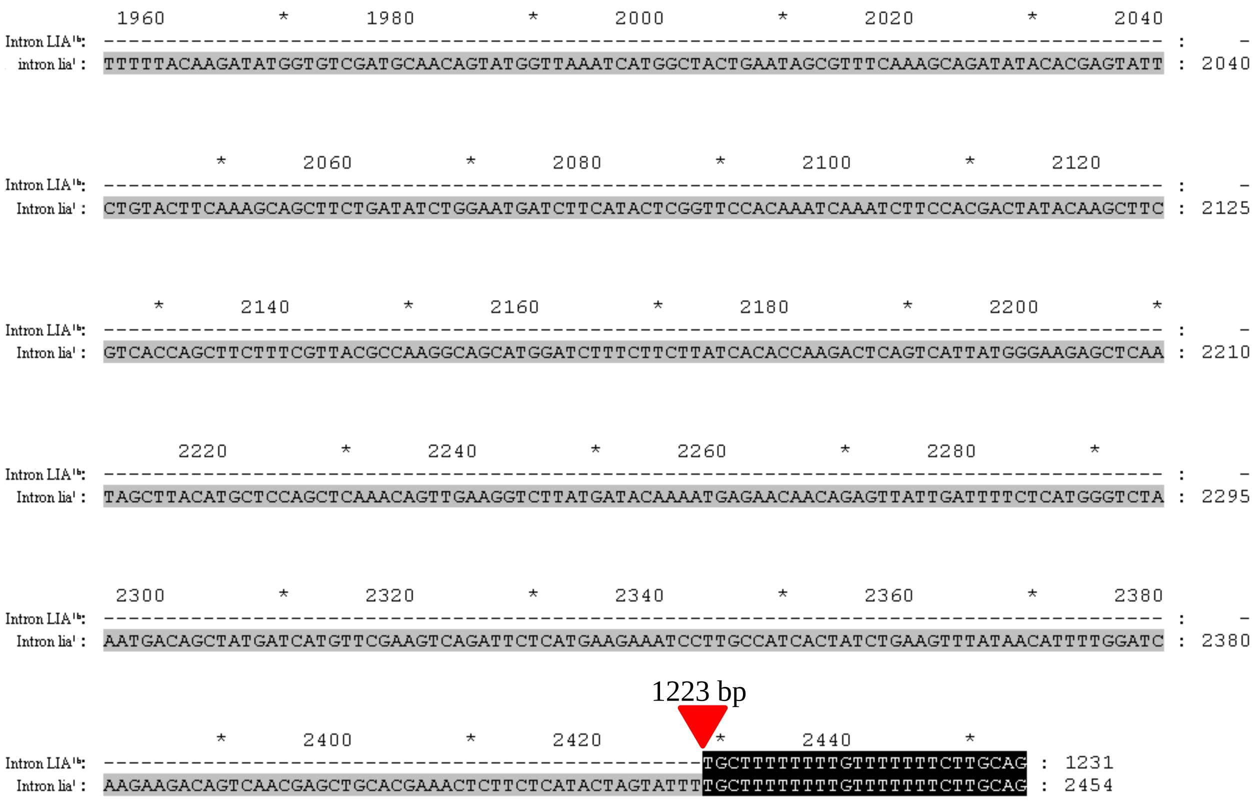
1960 * 1980 * 2000 * 2020 * 2040
intron lia[l] : TTTTTACAAGATATGGTGTCGATGCAACAGTATGGTTAAATCATGGCTACTGAATAGCGTTTCAAAGCAGATATACACGAGTATT : 2040
* 2060 * 2080 * 2100 * 2120
Intron lia[l] : CTGTACTTCAAAGCAGCTTCTGATATCTGGAATGATCTTCATACTCGGTTCCACAAATCAAATCTTCCACGACTATACAAGCTTC : 2125
* 2140 * 2160 * 2180 * 2200 *
Intron lia[l] : GTCACCAGCTTCTTTCGTTACGCCAAGGCAGCATGGATCTTTCTTCTTATCACACCAAGACTCAGTCATTATGGGAAGAGCTCAA : 2210
2220 * 2240 * 2260 * 2280 *
Intron lia[l] : TAGCTTACATGCTCCAGCTCAAACAGTTGAAGGTCTTATGATACAAAATGAGAACAACAGAGTTATTGATTTTCTCATGGGTCTA : 2295
2300 * 2320 * 2340 * 2360 * 2380
Intron lia[l] : AATGACAGCTATGATCATGTTCGAAGTCAGATTCTCATGAAGAAATCCTTGCCATCACTATCTGAAGTTTATAACATTTTGGATC : 2380
1223 bp
* 2400 * 2420 * 2440 *
Intron LIA[lb]: TGCTTTTTTTTGTTTTTTTCTTGCAG : 1231
Intron lia[l] : AAGAAGACAGTCAACGAGCTGCACGAAACTCTTCTCATACTAGTATTTTGCTTTTTTTTGTTTTTTTCTTGCAG : 2454

Supplemental Figure 8 Sequence alignment of the putative promoters (A) and exon 1 (B) of the *SalFAD2.LIA1* alleles *LIA[1a]*, *LIA[1b]* and *lia[1]* in yellow mustard.

A

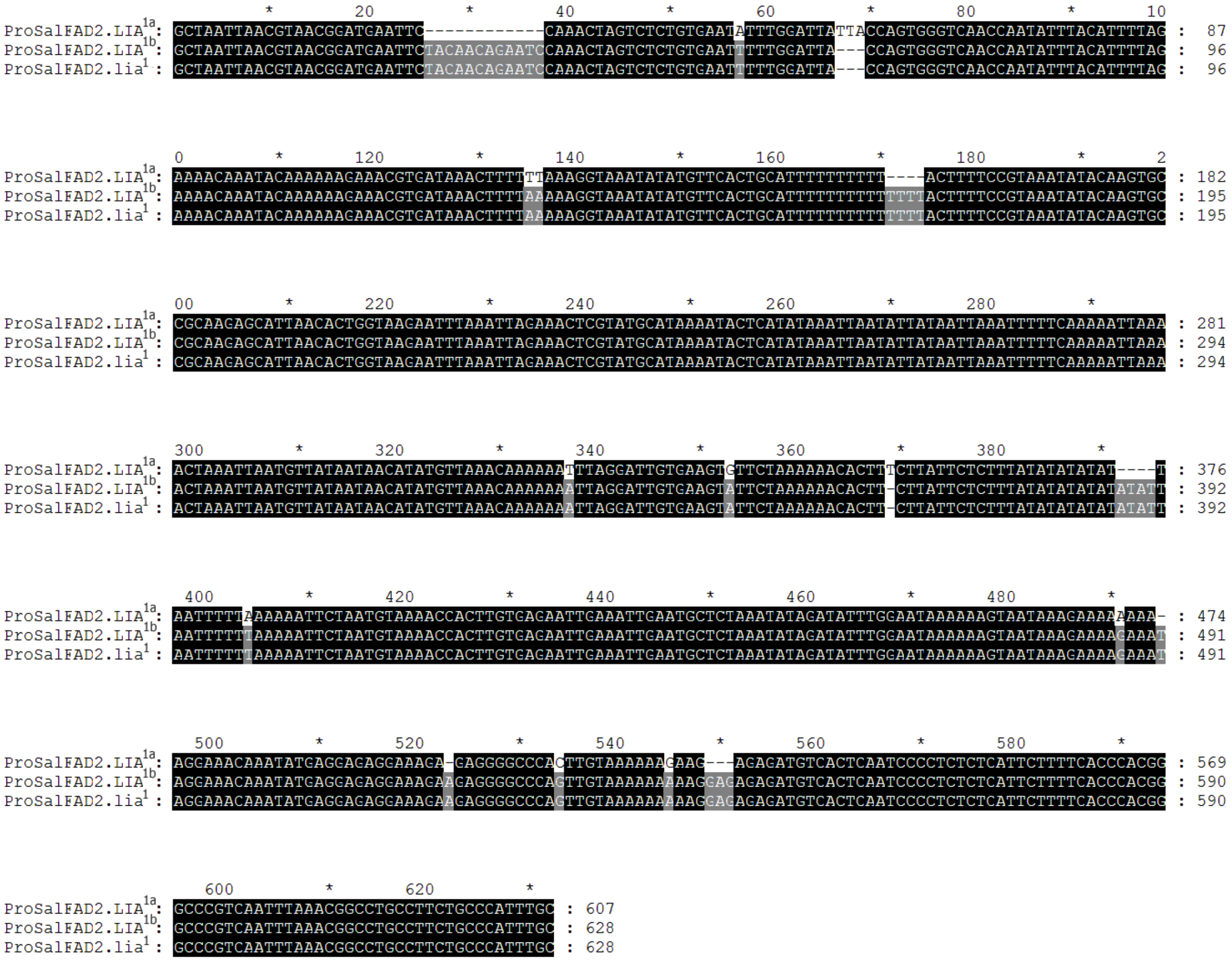

B

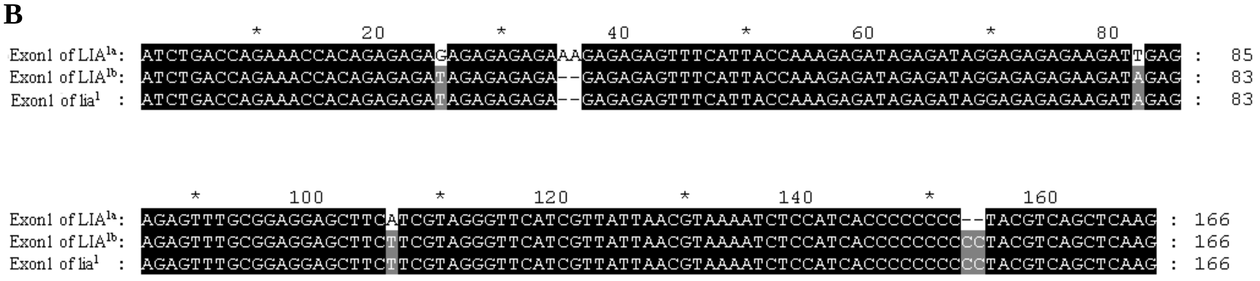

Supplemental Figure 9 Sequence alignment of the intron sequences of the *SalFAD2.LIA1* alleles *LIA*^{1a} and *LIA*^{1b}. Blue rectangle indicates the predicted motif "5UTR Py-rich stretch" in the intron of *LIA*^{1a}. Red stars indicate the point mutations at positions 868, 871 and 872 in the intron of *LIA*^{1b}.

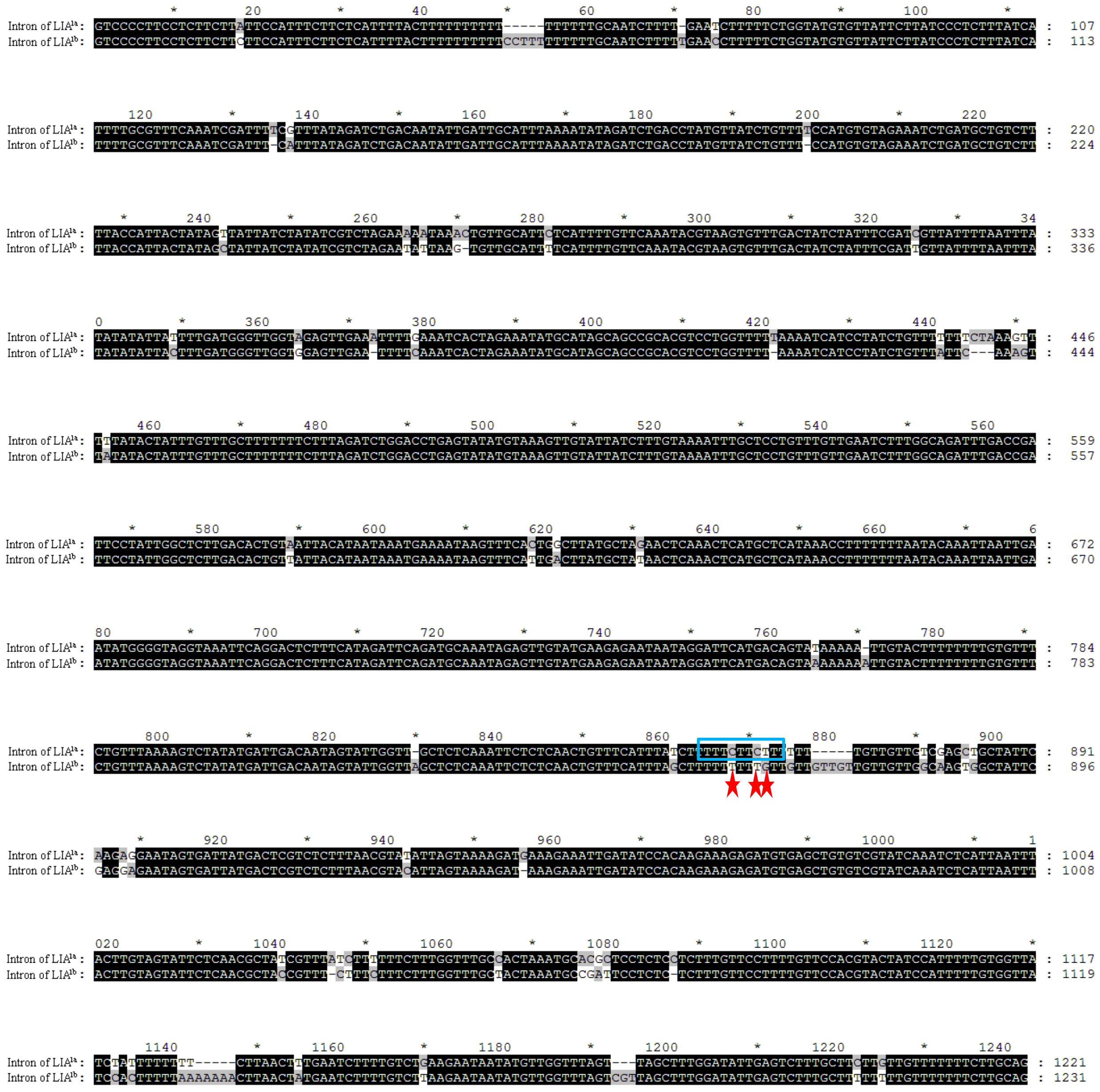

Supplemental Figure 10 Sequences analysis of the 1223 bp insertion in the intron of *lia*[1].

A. Nucleotide sequence of the 1223-bp insertion. Sequence annotation of the retrotransposon was predicted using the computer program GenScan (http://genes.mit.edu/GENSCAN.html). The coding region is highlighted in grey. 'GT' and 'AG' highlighted in yellow indicate the intron splicing site of the retrotransposon. The motif representing the primer binding site (PBS) was underlined and highlighted in green.

B. The 348 amino acids of the deduced protein of the retrotransposon predicted by the web-based computer program GenScan (http://genes.mit.edu/GENSCAN.html). The conserved domain is highlighted in blue.

A

ATTAGGTGTATAGTATTGTATACTTGTATATGAGTAAGTTAGAGTAGAGTAATACACACATGTATATATAGTGATC
ATTGTAACAACCTTTAATCTTAATGAAGATATTTCCATTTCATCGTCTTTCTGTTATGGTATCAGAGCGCATGAGCTG
PBS
AGCTCAGCGATTTCTCCGATCCGTTCTCCGATCATCGTCTTCATTCTTCCGATCATCATCTTCTTCATACCGTTCTTCG
ATCATCATCTCCGTTCTCCGATCATCATCTtCTGTtCACTTtCCTCCTCGGGTTCCTCATCATCTCAAGTCTTCcGCGCC
ATCTCGATTCCGTTTTATTTCTGATTTCATTTGATTCCCGATCTTCATCATGGGGAAGAGAAAAGTGTAATTTCGAA
GTACAGAAGCTCAACTCCCCAAGAAGCTTCAATCTCCGACTCATCGCGAATCGTCTCCAACCTCCGATCCTCGTCGC
GACGCATCCGGGATCTTCACATCAGGCTGTTCGTCTTCCTAATCTTGATAATCCAGTTGCTATTCGTTCTACTAAGCC
GTTTGAGGTATCCAACTCTCCAGATAACATACATAGTCCcATATTTTCTACATAGTTCGGATCATCCAGGCTTAGTG
CTCGCATCTGAGGCATTAGATGGAATGAACTATAGTATCTGGACCATTGCTATGACTACTAGCTTAGAGGCGAAGA
ACAAGCTTGGTTTTATTGATGGATCTATTGTTATGCCGGCTGAAACTGATCCTTTTTACAAGATATGGTGTCGATGC
AACAGTATGGTTAAATCATGGCTACTGAATAGCGTTTCAAAGCAGATATACACGAGTATTCTGTACTTCAAAGCAG
CTTCTGATATCTGGAATGATCTTCATACTCGGTTCCACAAATCAAATCTTCCACGACTATACAAGCTTCGTCACCAGC
TTCTTTCGTTACGCCAAGGCAGCATGGATCTTTCTTCTTATCACACCAAGACTCAGTCATTATGGGAAGAGCTCAAT
AGCTTACATGCTCCAGCTCAAACAGTTGAAGGTCTTATGATACAAAATGAGAACAACAGAGTTATTGATTTTCTCAT
GGGTCTAAATGACAGCTATGATCATGTTCGAAGTCAGATTCTCATGAAGAAATCCTTGCCATCACTATCTGAAGTTT
ATAACATTTTGGATCAAGAAGACAGTCAACGAGCTGCACGAAACTCTTCTCATACTAGTATTT

B

XDFSDPFSDHRLHSSDHHLLHTVLRSSSPFSDHHLLFTFLLGFLIISSLPRHLDSVLFLISFDSRSSSWGREKCNFEVQKLNSP
RSFNLRLIANRLQPPILVATHPGSSHQAVRLPNLDNPVAIRSTKPFEITYIVPYFLHSSDHPGLVLASEALDGMNYSIWTIA
MTTSLEAKNKLGFIDGSIVMPAETDPFYKIWCRCNSMVKSWLLNSVSKQIYTSILYFKAASDIWNDLHTRFHKSNLPRLY
KLRHQLLSLRQGSMDLSSYHTKTQSLWEELNSLHAPAQTVEGLMIQNENNRVIDFLMGLNDSYDHVRSQILMKKSLPS
LSEVYNILDQEDSQRAARNSSHTX

Supplemental Figure 11 Sequence alignments of the promoters, exon 1 and introns of the *SalFAD2.LIA2* alleles *LIA*^{2} and *lia*^{2}.

A. Sequence alignment of the putative promoters of *LIA*^{2} and *lia*^{2}. Red box indicates predicted transcription starting site. Blue box indicates the predicted “TATA-box” in *LIA*^{2}. Green box indicates the predicted “TATA-box” in *lia*^{2}.

B. Sequence alignment of the exon 1 of *LIA*^{2} and *lia*^{2}.

C. Sequence alignment of intron of *LIA*^{2} and *lia*^{2}.

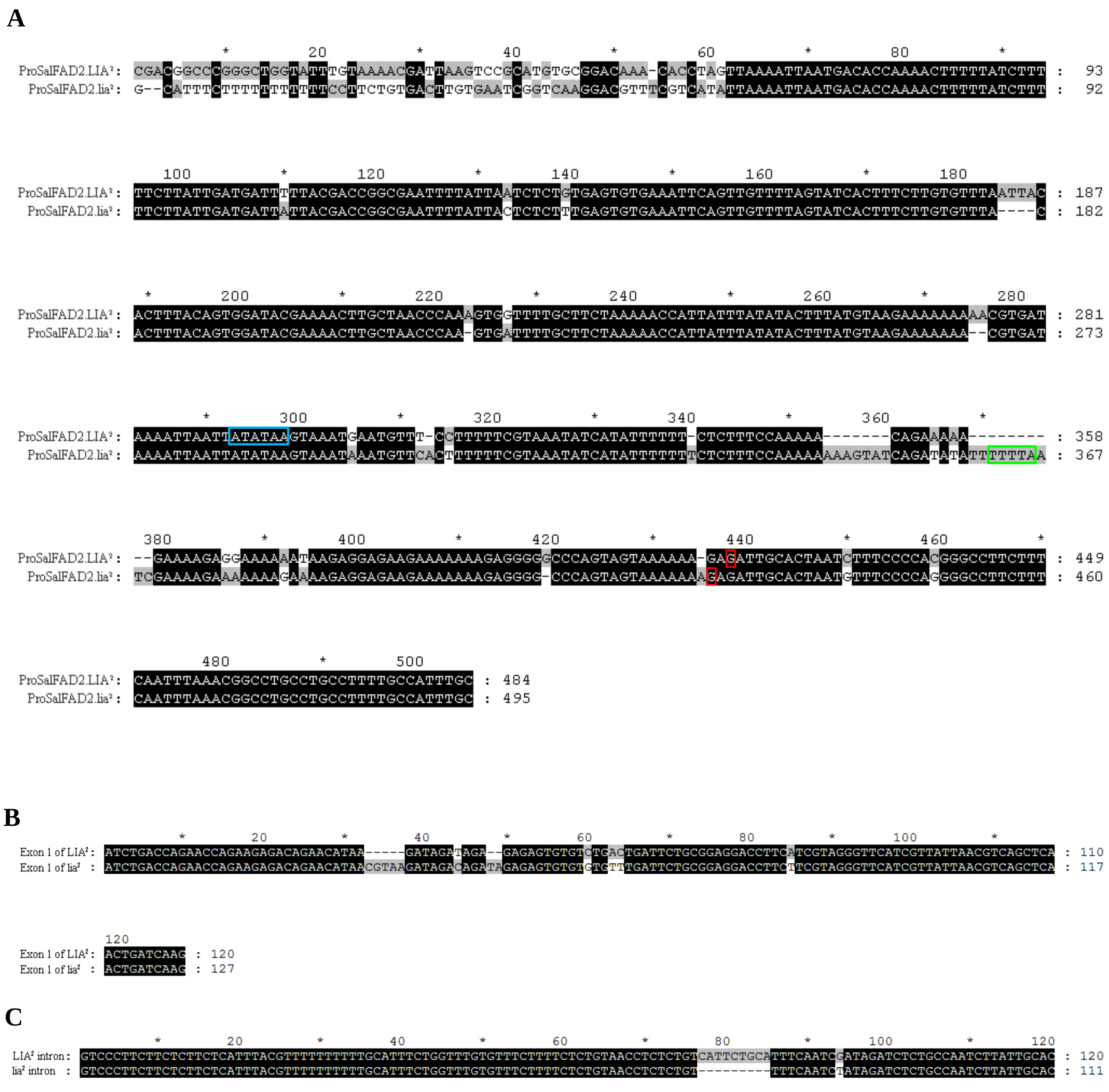

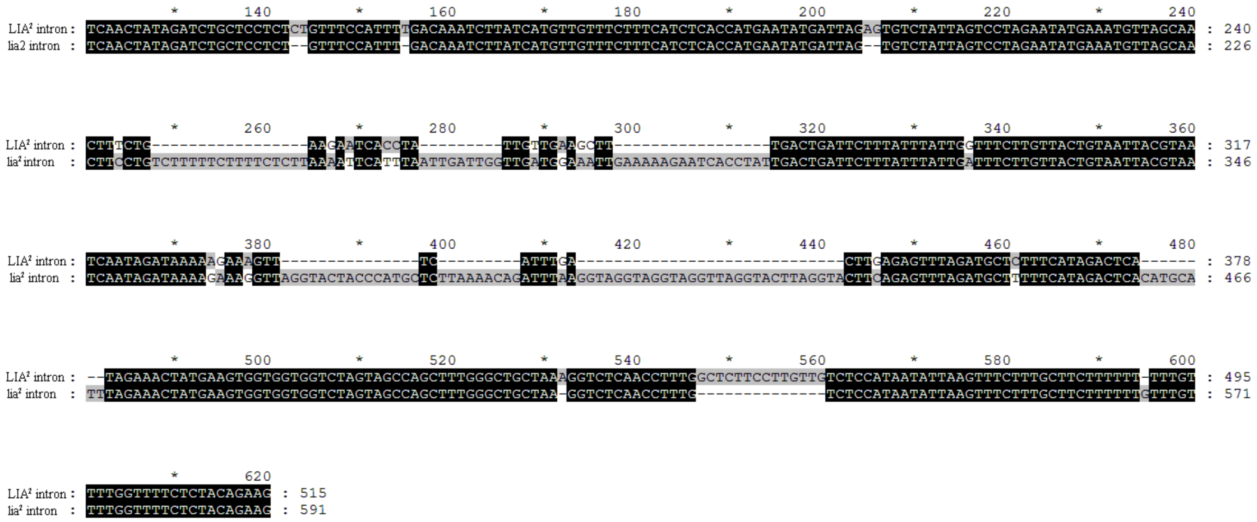
LIA² intron : TCAACTATAGATCTGCTCCTCTCTGTTTCCATTTTGACAAATCTTATCATGTTGTTTCTTTCATCTCACCATGAATATGATTAGAGTGTCTATTAGTCCTAGAATATGAAATGTTAGCAA : 240
lia2 intron : TCAACTATAGATCTGCTCCTCT--GTTTCCATTT-GACAAATCTTATCATGTTGTTTCTTTCATCTCACCATGAATATGATTAG--TGTCTATTAGTCCTAGAATATGAAATGTTAGCAA : 226
LIA² intron : CTTTCTG-----------------AAGAATCACCTA---------TTGTTGAAGCTT-----------------TGACTGATTCTTTATTTATTGGTTTCTTGTTACTGTAATTACGTAA : 317
lia² intron : CTTCCTGTCTTTTTCTTTTCTCTTAAAATTCATTTAATTGATTGGTTGATGGAAATTGAAAAAGAATCACCTATTGACTGATTCTTTATTTATTGATTTCTTGTTACTGTAATTACGTAA : 346
LIA² intron : TCAATAGATAAAAAGAAAGTT---------------TC---------ATTTGA-----------------------------CTTGAGAGTTTAGATGCTCTTTCATAGACTCA------ : 378
lia² intron : TCAATAGATAAAAGAAAGGTTAGGTACTACCCATGCTCTTAAAACAGATTTAAGGTAGGTAGGTAGGTTAGGTACTTAGGTACTTCAGAGTTTAGATGCTTTTTCATAGACTCACATGCA : 466
LIA² intron : --TAGAAACTATGAAGTGGTGGTGGTCTAGTAGCCAGCTTTGGGCTGCTAAAGGTCTCAACCTTTGGCTCTTCCTTGTTGTCTCCATAATATTAAGTTTCTTTGCTTCTTTTTT-TTTGT : 495
lia² intron : TTTAGAAACTATGAAGTGGTGGTGGTCTAGTAGCCAGCTTTGGGCTGCTAA-GGTCTCAACCTTTG--------------TCTCCATAATATTAAGTTTCTTTGCTTCTTTTTTGTTTGT : 571
LIA² intron : TTTGGTTTTCTCTACAGAAG : 515
lia² intron : TTTGGTTTTCTCTACAGAAG : 591

Supplemental Figure 12 Sequence alignments of the putative promoter and introns of the *SalFAD2.LIA1* allele LIA^{1a} and the *SalFAD2.LIA2* allele LIA^{2}.

A. Sequence alignment of the putative promoters of LIA^{1a} and LIA^{2}.

B. Sequence alignment of the introns of LIA^{1a} and LIA^{2}. Blue box indicates the predicted transcription starting site at position 825 in the intron of LIA^{1a}. Red box indicates the predicted "TATA-box" TTTAAAA at position 787 in the intron of LIA^{1a}.

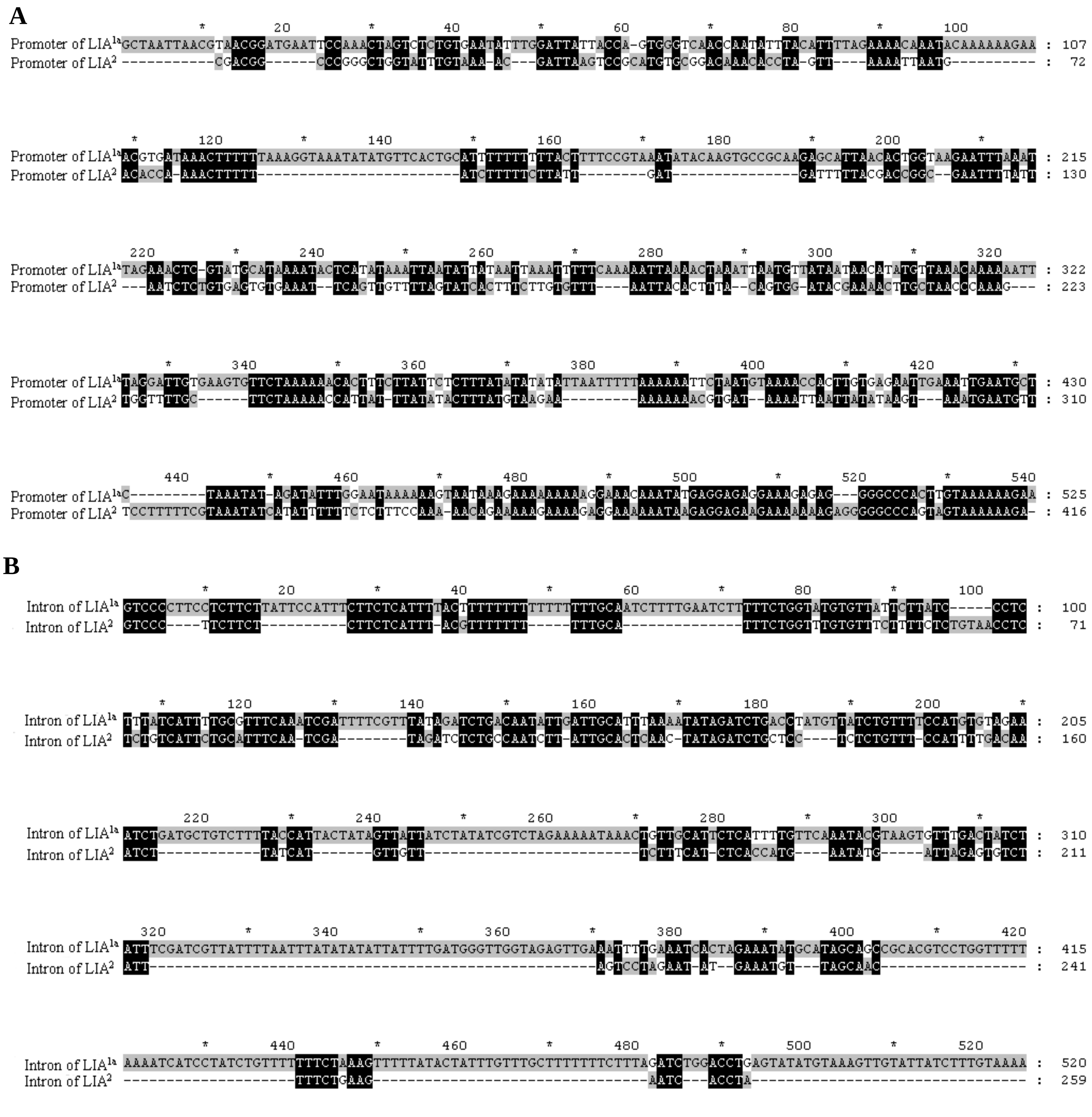

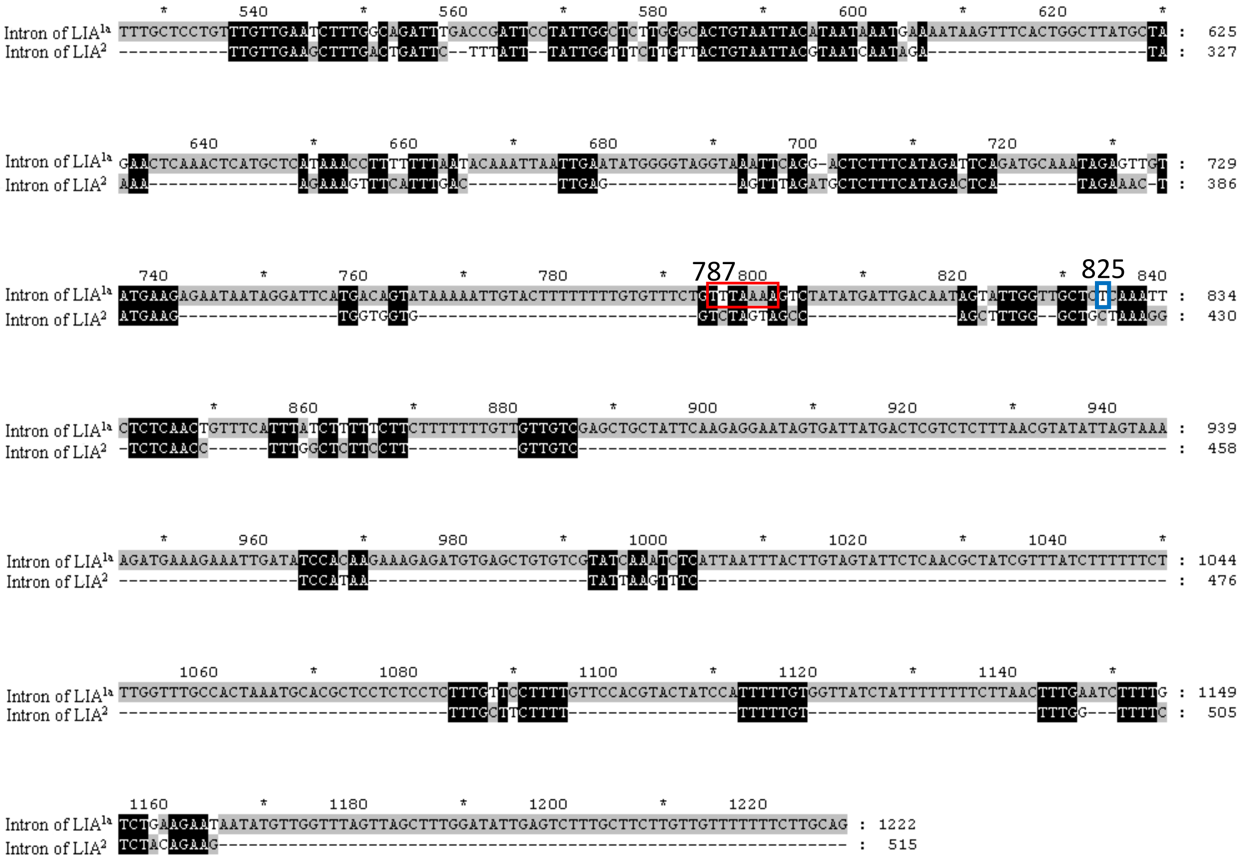
787
825

Supplemental Table 1 Primer pairs used in this study

No.	Name	Sequence (5'-3')	Purpose
1	FAD2P1L	ATGGGTGCAGGTGGAAGAATG	To clone the entire coding region of *LIA*^{1a}, *LIA*^{1b}, *lia*^{1}, *LIA*^{2} and *lia*^{2}
	FAD2P1R	CTTCACCATCATCCTCATAAC	
2	DGSP1-C2	GTTCTCCACGATGCCGCATTATCACGC	To clone the 3' downstream sequences from the translation stop coding site for *LIA*^{1a}, *LIA*^{1b}, *lia*^{1}, *LIA*^{2} and *lia*^{2}
	DGSP2-C2	TATTATCAGTTCGATGGAACACCAGTG	
3	LIA^{1}1stRGSP1	AGCCGAGAGTGAACTGAACAGTTAACA	1st round PCR walking to clone 5' upstream sequence of *LIA*^{1a}, *LIA*^{1b}and *lia*^{1}
	LIA^{1}1stRGSP2	GTACGTCCAAGAGGGTTGTTGAGGTAT	
4	LIA^{1}2ndRGSP1	ACGTGGAACAAAAGGAACAAAGAGAGAG	2nd round PCR walking to clone further 5' upstream sequence of *LIA*^{1a}, *LIA*^{1b} and *lia*^{1}
	LIA^{1}2ndRGSP2	TGATACGACACAGCTCACATCTCTTTCT	
5	PRO-L1	GCTAATTAACGTAACGGATG	To clone the putative promoter sequences of *LIA*^{1a}, *LIA*^{1b} and *lia*^{1}
	PRO-R1	TGTGAGACGATTGAGTGACA	
6	LIA^{1}GAP-L	GCTAATTAACGTAACGGATG	To clone the DNA fragment between the promoter and 5' flanking sequence of *LIA*^{1a}, *LIA*^{1b}and *lia*^{1}
	LIA^{1}GAP-R	GTGTCAAGAGCCAATAGGAA	
7	LIA^{2}1stRGSP1	AGCCGAGAGTGAACTGAACAGTTAACA	1st round PCR walking to clone 5' upstream sequence of *LIA*^{2}and *lia*^{2}
	LIA^{2}1stRGSP2	CCAAGAGGGTTGTTGTTGAGGTACT	
8	LIA^{2}2ndRGSP1	TTGAGCTGACGTTAATAACGATGAACC	2nd round PCR walking to clone further 5' upstream sequence of *LIA*^{2}and *lia*^{2}
	LIA^{2}2ndRGSP2	CTTATGTTCTGTCTCTTCTGGTTCTGG	
9	LIA^{1}W-L	GCTAATTAACGTAACGGATG	To clone the entire gene sequences of *LIA*^{1a}, *LIA*^{1b} and *lia*^{1}
	LIA^{1}W-R	CTGTCCGGTTCTACATAGAT	
10	LIA^{2}W-L	GCTCAACTGATCAAGAAGAA	To clone the entire gene sequences of *LIA*^{2}and *lia*^{2}
	LIA^{2}W-R	CTGTCCGGTTCTACATAGAT	
11	LIA^{1}WC-L	AACCAGAGAGAGAGAGAGAG	To clone the cDNA for *LIA*^{1a}, *LIA*^{1b} and *lia*^{1}
	LIA^{1}WC-R	CTTCACCATCATCCTCATAAC	

12	LIA^2WC-L	GCTCAACTGATCAAGAAGAA	To clone the cDNA for *LIA^2* and *lia^2*
	LIA^2WC-R	CTTCACCATCATCCTCATAAC	
13	$YLIA^1CDS$-L	GTTATGGGTGCAGGTGGAAGAAT	To amplify the coding regions of *LIA^{1a}* and *LIA^{1b}* (*lia^1*) for yeast expression
	$YLIA^1CDS$-R	GCATCATCATCATCATCC	
14	$YLIA^2CDS$-L	GCTCAACTGATCAAGAAGAA	To amplify the coding regions of *LIA^2* and *lia^2* for yeast expression
	$YLIA^2CDS$-R	ACTCAACTTATTATTGTACCAGAAC	
15	LIA^1RT-L	AACCAGAGAGAGAGAGAGAG	RT-PCR for *LIA^{1a}*, *LIA^{1b}* and *lia^1*
	LIA^1RT-R	CGTCGTAGTAAGGTCTTCCG	
16	LIA^2RT-L	GCTCAACTGATCAAGAAGAA	RT-PCR for *LIA^2*and *lia^2*
	LIA^2RT-R	AGGGTTGTTGAGGTATTTGC	
17	LIA^1QRT-L	AACCAGAGAGAGAGAGAGAG	Quantitative Real-time PCR for *LIA^{1a}*, *LIA^{1b}* and *lia^1*
	LIA^1QRT-R	TTCTTGAGCTGACGTAGG	
18	LIA^2QRT-L	GAGTGTGTCTGACTGATTCT	Quantitative Real-time PCR for *LIA^2*and *lia^2*
	LIA^2QRT-R	GAAGGAGTAACTTGCATTCT	
19	ActinF	ACGAGCTACCTGACGGACAAG	RT-PCR, Quantitative Real-time PCR
	ActinR	GAGCGACGGCTGGAAGAGTA	
20	$LIA^{1a}PRO$-L	CGAAGCTTGCTAATTAACGTAACGGATG	To clone the 693 bp promoter sequence of *LIA^{1a}* for promoter function analysis
	$LIA^{1a}PRO$-R	GGTCTAGATCTCAATCTTCTCTCTCCTA	
21	$LIA^{1b}PRO$-L	CGAAGCTTGCTAATTAACGTAACGGATG	To clone the 714 bp promoter sequence of *LIA^{1b}* for promoter function analysis
	$LIA^{1b}PRO$-R	GGTCTAGATCTCAATCTTCTCTCTCCTA	
22	LIA^2PRO-L	CCAAGCTTAACGATTAAGTCCGCATGTG	To clone the 566 bp promoter sequence of *LIA^2* for promoter function analysis
	LIA^2PRO-R	GCTCTAGAGCTGACGTTAATAACGATGA	
23	lia^2PRO-L	GCAAGCTTACTCCGTATCACGTTCTAG	To clone the 921 bp promoter sequence of *lia^2* for promoter function analysis
	lia^2PRO-R	CCTCTAGACAGTTGAGCTGACGTTAATA	
24	$LIA^{1a}Intron$-L	TACTGCAGAGATAGGAGAGAGAAGATTG	To clone the 1286 bp intron sequence of *LIA^{1a}* for function analysis

	LIA[1a]Intron-R	ATGGATCCCCAAAGCTAACTAAACCAAC	
25	LIA[1b]Intron-L	TACTGCAGAACCACAGAGAGATAGAGAG	To clone the 1354 bp intron sequence of LIA^{1b} for function analysis
	LIA[1b]Intron-R	ATGGATCCCAATATCCAAAGCTAACGAC	
26	LIA[2]Intron-L	TACTGCAGGAACCAGAAGAGACAGAACA	To clone the 579 bp intron sequence of LIA^2 for function analysis
	LIA[2]Intron-R	ATGGATCCTTATGGAGACAACAAGGAAG	
27	lia[2]Intron-L	TACTGCAGACCAGAACCAGAAGAGACAG	To clone the 617 bp intron sequence of lia^2 for function analysis
	lia[2]Intron-R	AAGGATCCTAGACCACCACCACTTCATA	
28	Intron-L	CTTCTTCGTAGGGTTCATCG	To produce allele specific marker for LIA^{1a}, LIA^{1b}, lia^1, LIA^2 and lia^2
	Intron-R	AACCTGCATTCTTCCACCT	
29	LPRO-L	CGGATGAATTCTACAACAG	To generate marker specific for LIA^{1b}
	LPRO-R	TTGAGTGACATCTCTCTCCT	
30	FAD3-L1	GCCATGTTGAAAACGACGAG	To produce co-dominant markers specific for *SalFAD3.LA1* and *SalFAD3.LA2*
	FAD3-R1	ACAGTGTATCTGAGCATCCG	
31	FAD3-L2	ATGGTTGTYGCTATGGACC	To generate a marker specific to *SalFAD3.LA1* in Y1798 × Y1801
	FAD3-R2	ACAGTGTATCTGAGCATCCG	
32	FAE1-L1	CAATGTGTATGCCTGCATGA	To generate marker specific for *FAE1* alleles E^1 and E^2 in the F2 population of Y1798 × Y1801
	FAE1-R1	CGAGTATCGAGCTATGTCTG	
33	FAE1-L2	ATGACGTCCGTTAACGTA	To generate marker specific for *FAE1* alleles E^1 and *e* in the F2 population of Y1798 × Y514
	FAE1-R2	AAGGTGCCAGAACACTGCTC	

Supplementary Table 2. Summary of QTLs for C18:2, C18:3 and C22:1 contents in the F_2 populations derived from the three crosses Y1798 (low) × Y1801 (high), Y1798 (low) × Y514 (medium) and Y1801 (high) × Y514 (medium)

Crosses	Fatty acid	Linkage groups	Gene co-localized with the QTL	Position (cM)	LOD score	$R^{2\ a}$
Y1798 (low) × Y1801 (high)	C18:2	Sal01	*SalFAD2.LIA1*	59.3	40.63	43.2
		Sal02	*SalFAD3.LA1*	30.7	40.44	42.8
		Sal03	*FAE1*	30.4	9.99	5.6
	C18:3	Sal02	*SalFAD3.LA1*	30.7	38.30	67.6
		Sal03	*FAE1*	26.8	8.97	8.2
	C22:1	Sal03	*FAE1*	30.4	37.10	73.1
Y1798 (low) × Y514 (medium)	C18:2	Sal01	*SalFAD2.LIA1*	49.4	25.44	47.3
		Sal03	*FAE1*	53.0	7.36	24.4
		Sal08	*SalFAD2.LIA2*	59.8	3.47	9.5
	C18:3	Sal01	*SalFAD2.LIA1*	52.5	4.07	9.0
		Sal02	*SalFAD3.LA1*	33.5	3.27	7.3
		Sal03	*FAE1*	51.6	12.50	38.2
	C22:1	Sal03	*FAE1*	50.6	56.90	89.0
Y1801(high) × Y514 (medium)	C18:2	Sal02	*SalFAD3.LA1*	39.9	14.40	42.6
		Sal01	*SalFAD2.LIA1*	47.7	3.54	14.9
	C18:3	Sal02	*SalFAD3.LA1*	39.9	30.10	67.6
		Sal10	*SalFAD3.LA2*	17.5	8.40	8.7
	C22:1	Sal03	*FAE1*	61.9	35.60	66.2

[a] Percentage of the total phenotypic variation explained by QTL.

Supplemental Table 3 The C18:3 and C22:1 genotypes of the parental lines Y1798, Y514 and Y1801 were determined using the *FAD3* genes, *SalFAD3.LA1* and *SalFAD3.LA2*, and *FAE1* gene allele-specific markers, respectively.

Parental lines	*FAD3* genotypes		*FAE1* genotypes
	SalFAD3.LA1	*SalFAD3.LA2*	
Y1798	$LA^{1a}LA^{1a}$	$LA^{2}LA^{2}$	$E^{1}E^{1}$
Y514	$LA^{1}LA^{1}$	$la^{2}la^{2}$	*ee*
Y1801	$la^{1}la^{1}$	$LA^{2}LA^{2}$	$E^{2}E^{2}$
